# Molecular basis for functional diversity among microbial Nep1-like proteins

**DOI:** 10.1371/journal.ppat.1007951

**Published:** 2019-09-03

**Authors:** Tea Lenarčič, Katja Pirc, Vesna Hodnik, Isabell Albert, Jure Borišek, Alessandra Magistrato, Thorsten Nürnberger, Marjetka Podobnik, Gregor Anderluh

**Affiliations:** 1 Department of Molecular Biology and Nanobiotechnology, National Institute of Chemistry, Hajdrihova, Ljubljana, Slovenia; 2 Department of Biology, Biotechnical Faculty, University of Ljubljana, Jamnikarjeva, Ljubljana, Slovenia; 3 Center of Plant Molecular Biology (ZMBP), Eberhard-Karls-University Tübingen, Auf der Morgenstelle, Tübingen, Germany; 4 CNR-IOM-Democritos at International School for Advanced Studies (SISSA), Trieste, Italy; 5 Department of Biochemistry, University of Johannesburg, Auckland Park, South Africa; John Innes Centre, UNITED KINGDOM

## Abstract

Necrosis and ethylene-inducing peptide 1 (Nep1)-like proteins (NLPs) are secreted by several phytopathogenic microorganisms. They trigger necrosis in various eudicot plants upon binding to plant sphingolipid glycosylinositol phosphorylceramides (GIPC). Interestingly, HaNLP3 from the obligate biotroph oomycete *Hyaloperonospora arabidopsidis* does not induce necrosis. We determined the crystal structure of HaNLP3 and showed that it adopts the NLP fold. However, the conformations of the loops surrounding the GIPC headgroup-binding cavity differ from those of cytotoxic *Pythium aphanidermatum* NLP_Pya_. Essential dynamics extracted from μs-long molecular dynamics (MD) simulations reveals a limited conformational plasticity of the GIPC-binding cavity in HaNLP3 relative to toxic NLPs. This likely precludes HaNLP3 binding to GIPCs, which is the underlying reason for the lack of toxicity. This study reveals that mutations at key protein regions cause a switch between non-toxic and toxic phenotypes within the same protein scaffold. Altogether, these data provide evidence that protein flexibility is a distinguishing trait of toxic NLPs and highlight structural determinants for a potential functional diversification of non-toxic NLPs utilized by biotrophic plant pathogens.

## Author summary

The rhizosphere and phyllosphere of terrestrial plants are home to a number of microorganisms, many of which are potentially pathogenic at certain stages during their associations with plants. The pathogens use diverse routes to penetrate physical barriers and colonize host plants with different lifestyles. Necrosis and ethylene inducing peptide 1 (Nep1)-like proteins (NLPs) are widely distributed among prokaryotic and eukaryotic organisms, like fungi, bacteria and oomycetes, and may infect a range of different crops, such as potato, tomato, soybean and tobacco, thus posing major threat to agriculture worldwide. It was shown that NLPs function as cytolytic toxins that induce plasma membrane leakage by binding to plant membrane sphingolipid receptor. Interestingly, non-toxic NLP proteins can also be secreted by several hemibiotrophic and obligate biotrophic pathogens. This study provides a structural and functional characterization of a non-cytotoxic HaNLP3 protein from *Hyaloperonospora arabidopsidis* that could explain how these proteins evolved a range of new functions in variety of pathogens with different lifestyles.

## Introduction

Necrosis- and ethylene-inducing peptide 1-like proteins (NLPs) have been found in plant-associated prokaryotic (bacteria) and eukaryotic (fungi, oomycetes) microorganisms and are widely known to induce necrosis and ethylene production in eudicot plants [1–3]. NLPs share a conserved necrosis inducing protein (NPP1) domain, typically containing the heptapeptide motif, GHRHDWE, which is crucial for toxicity. NLPs are divided into five phylogenetic group types based on comparative and functional analysis of NLP sequences: fungal/bacterial type 1, oomycete type 1, oomycete type 1a, type 2 and type 3 [4, 5]. Type 1 NLPs contain a conserved disulfide bond and are found predominately in microorganisms that are associated with plants. Several NLPs produced by oomycetes lack a complete heptapeptide motif and, as a result, are non-toxic. These proteins cluster together and form group type 1a. Type 2 NLPs are distinct from type 1 NLPs due to the presence of a second conserved disulfide bond and an additional putative calcium-binding domain. Little is known about type 3 NLPs; however, it is predicted that they contain three disulfide bonds and a distinct overall structure [5].

The first crystal structure of a toxic type 1 NLP from the oomycete *Pythium aphanidermatum* (NLP_Pya_) revealed a central β-sandwich surrounded by α-helices [6]. MpNEP2 from the fungus *Moniliophthora perniciosa* exhibited a similar structure [7]. Comparison of three-dimensional models revealed structural similarity of the NLP family fold to actinoporins [6]. Actinoporins constitute a family of cytolytic proteins that act by disrupting the integrity of target cell membranes through pore formation after specific binding to sphingomyelin [8–10]. The structural similarity suggested that NLPs employ a similar cytolytic mechanism as actinoporins. Recently, we reported that glycosylinositol phosphorylceramides (GIPC), the major class of plant sphingolipids, represent target molecules for NLP binding to plant plasma membranes [11]. GIPCs consist of inositol phosphorylceramide covalently bound to glucuronic acid and a variable number of terminal hexoses [12]. The type and number of hexose moieties vary highly between plants and plant tissues, with eudicots typically bearing two terminal sugars (series A) and monocots three terminal sugars (series B) [12, 13]. In Lenarčič *et al*. (2017) we showed that binding of the GIPC terminal hexose moiety to NLP_Pya_ induces several conformational changes within the toxin that may precede membrane attachment and host cell lysis. Similar to actinoporins, the interaction of NLP_Pya_ with the membrane is stabilized via a tryptophan residue in the loop adjacent to the sphingolipid binding site. Importantly, monocots are unaffected by NLP proteins because their plasma membranes mostly lack series A GIPCs [11].

*Hyaloperonospora arabidopsidis*, a downy mildew pathogen of *Arabidopsis*, is an obligate biotrophic oomycete [14]. *H*. *arabidopsidis* secretome analysis revealed NLPs as one of many secreted protein families [15]. None of the ten secreted HaNLPs were found to be phytotoxic [15]. Based on amino acid sequence comparison, HaNLP3 is the most similar to prototypical toxic oomycete type I NLPs. Despite having a conserved heptapeptide motif and being expressed during *Arabidopsis* infection, HaNLP3 does not induce necrosis on plant cells [6, 15]. The role of HaNLPs during biotrophic infection remains to be determined; however, they represent an excellent model system to study NLP proteins with regards to cytolytic activity toward plant cells, and, more generally, evolution and diversification within the NLP superfamily.

The aim of the current study was to provide structural and functional information on similarities and differences between non-toxic HaNLP3 and toxic NLP_Pya_, both members of the oomycete type 1 NLP group. A detailed comparison of the two protein structures allowed us to assign a functional role to specific protein structural elements. These findings demonstrate how differences within the same protein scaffold fine-tune functional plasticity of NLP proteins, further illuminating the mechanistic mode of action of toxic NLPs.

## Results

### Non-toxic HaNLP3 protein does not bind to GIPCs

**Fig 1:**
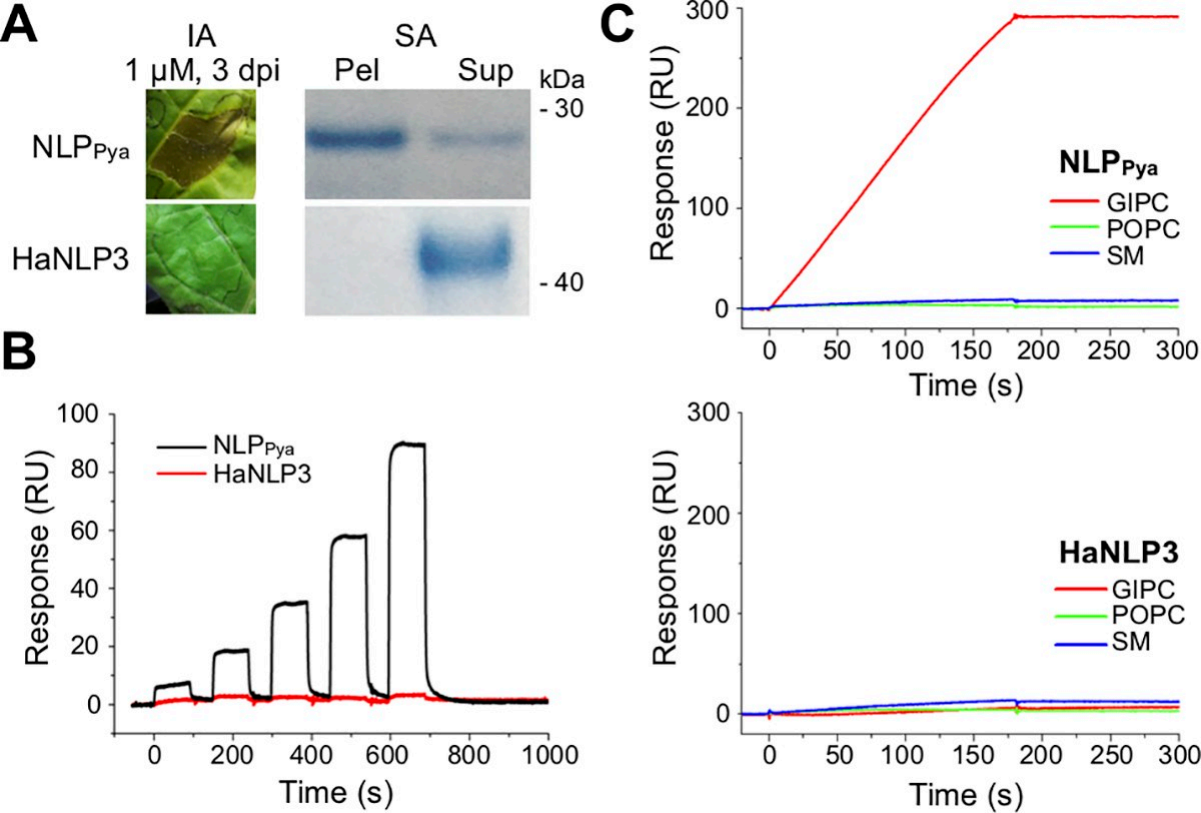
GIPC-binding properties of non-toxic HaNLP3 in comparison to toxic NLP_Pya_. (A) HaNLP3 and NLP_Pya_-induced tobacco leaf necrosis and binding of proteins to POPC:GIPCs multilamellar vesicles as revealed by infiltration assay (IA) and liposome sedimentation assay (SA), respectively. Dpi, days post-infiltration; Pel, pellet; Sup, supernatant. (B) Surface plasmon resonance analysis of HaNLP3 and NLP_Pya_ binding to immobilized lipid bilayers composed of POPC:GIPCs. (C) Binding of GIPCs, POPC or sphingomyelin (SM) to immobilized NLP_Pya_ and HaNLP3.

First, we confirmed the inability of recombinant HaNLP3 to induce necrosis of tobacco plant cells [15] (Fig 1A). We next checked whether the difference in cytotoxicity between NLP_Pya_ and HaNLP3 is due to inability of the latter to bind the headgroup of plant sphingolipids. As opposed to toxic NLP_Pya_, HaNLP3 was unable to bind to multilamellar vesicles composed of 1-palmitoyl-2-oleoyl-*sn*-glycero-3-phosphocholine (POPC) and tobacco leaf GIPCs (POPC:GIPCs) (Fig 1A). Surface plasmon resonance (SPR) experiments on immobilized lipid bilayers containing GIPCs revealed concentration-dependent binding of NLP_Pya_ but not HaNLP3 (Fig 1B). In addition, HaNLP3 immobilized on the surface of the sensor chip was not able to bind GIPCs as compared to NLP_Pya_ (Fig 1C). Importantly, neither HaNLP3 nor NLP_Pya_ bind POPCs or sphingomyelin (Fig 1C).

### Structure of HaNLP3 reveals unique features of the NLP fold

**Fig 2:**
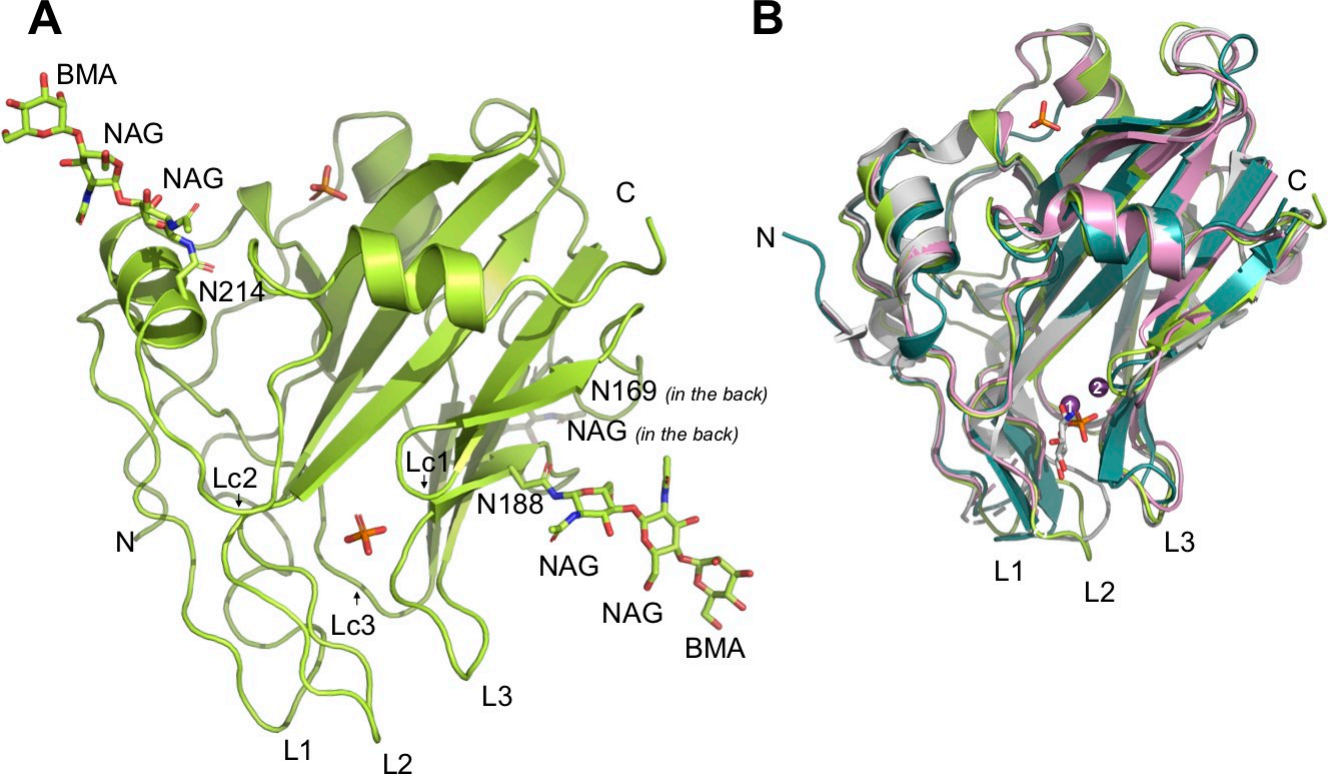
Atomic structure of non-toxic HaNLP3. (A) Overall crystal structure of HaNLP3. Glycosylation sites (N169, N188 and N214) are highlighted and sugar moieties shown as sticks. Phosphate ions are displayed as orange and red sticks. NAG, N-acetylglucosamine; BMA, mannose. (B) Superposition of HaNLP3 (green), apo-NLP_Pya_ (PDB-ID 3GNZ; white), NLP_Pya_-glucosamine complex (PDB-ID 5NNW; pink) and MpNEP2 structures (PDB-ID 3ST1; cyan). Mg^2+^ ions are displayed as purple spheres: (1) position of Mg^2+^ in apo-NLP_Pya_; (2) position of Mg^2+^ in NLP_Pya_-glucosamine complex. Glucosamine (white) and phosphate ions (orange and red) are shown in sticks.

To investigate structural differences between non-toxic HaNLP3 and toxic NLPs, the HaNLP3 crystal structure was determined (Fig 2A, S1 Fig, Table 1). The HaNLP3 fold highly resembles those of toxic homologs (Fig 2B): apo-NLP_Pya_ [6], NLP_Pya_-glucosamine complex [11] and MpNEP2 [7]. The HaNLP3 structure comprises a nine-stranded β-sandwich flanked by three α-helices (Fig 2). RMSD values, as given by DALI [18], between all C_α_ atoms of HaNLP3 and apo-NLP_Pya_ (PDB ID 3GNZ), NLP_Pya_ in complex with glucosamine (PDB ID 5NNW) and MpNEP2 (PDB ID 3ST1) are 1.1 Å, 1.1 Å and 1.5 Å, respectively. The fold of the conserved loops of NLP proteins, L1-L3, is well defined in HaNLP3 (Fig 2A). All NLP structures determined so far share a crevice located between loops L2 and L3, which was recently shown to be the GIPC receptor binding motif within toxic NLPs (Fig 2B) [11]. In addition to L2 and L3, the crevice is also lined with loops Lc1-Lc3; the same configuration is found in HaNLP3 (Fig 2A). The structure of HaNLP3 is defined in electron density from residue His28 to residue Thr242, and is thus missing 27 N-terminal and 5 C-terminal residues. Three glycosylated sites were observed at Asn169, Asn188 and Asn214 (Fig 2A). In addition, two bound phosphate ions were observed, likely due to the crystallization conditions (Fig 2A).

**Table 1:** Data collection and refinement statistics.

	HaNLP3	NLP_Pya_^P41A, D44N, N48E^
**Data collection**		
Space group	P2_1_2_1_2_1_	P4_1_2_1_2
Cell dimensions		
*a*, *b*, *c* (Å)	44.7, 48.9, 112.3	87.4, 87.4, 116.0
(°)	90.0, 90.0, 90.0	90.0, 90.0, 90.0
Resolution (Å)	44.82–2.00	42.29–1.95
*R*_meas_ (%)	18.9 (33.0)	23.4 (74.6)
Mean of *I* / σ*I*	8.5 (5.4)	9.4 (3.5)
CC_1/2_ (%)	97.9 (94.2)	99.1 (92.5)
Completeness (%)	94.0 (99.3)	100.0 (100.0)
Redundancy	6.2 (6.4)	13.0 (12.2)
No. unique reflections	16264	33415
**Refinement**		
Resolution (Å)	41.51–2.00	42.28–1.95
No. reflections	16262	33411
*R*_work_ / *R*_free_ (%)	18.2 / 21.1	17.0 / 20.7
No. atoms		
Protein	1687	3168
N-acetylglucosamine (glycosylation)	70	/
Mannose (glycosylation)	22	/
Ions	10 (PO_4_^3-^)	3 (Mg^2+^)
Water	136	414
*B*-factors (Å^2^)		
Protein	22.4	16.8
N-acetylglucosamine (glycosylation)	41.4	/
Mannose (glycosylation)	53.5	/
Ions	36.7 (PO_4_^3-^)	24.3 (Mg^2+^)
Water	28.8	21.4
R.m.s. deviations		
Bond lengths (Å)	0.008	0.007
Bond angles (°)	1.116	0.759
Ramachandran plot		
Favored (%)	94.4	97.0
Allowed (%)	5.6	3.0
Disallowed (%)	0.0	0.0

Each dataset was collected from a single crystal. Values in parentheses are for the highest-resolution shell. Crystal parameters and data collection statistics were derived from XDS [16]. Refinement statistics and Ramachandran analysis were obtained from Phenix [17].

**Fig 3:**
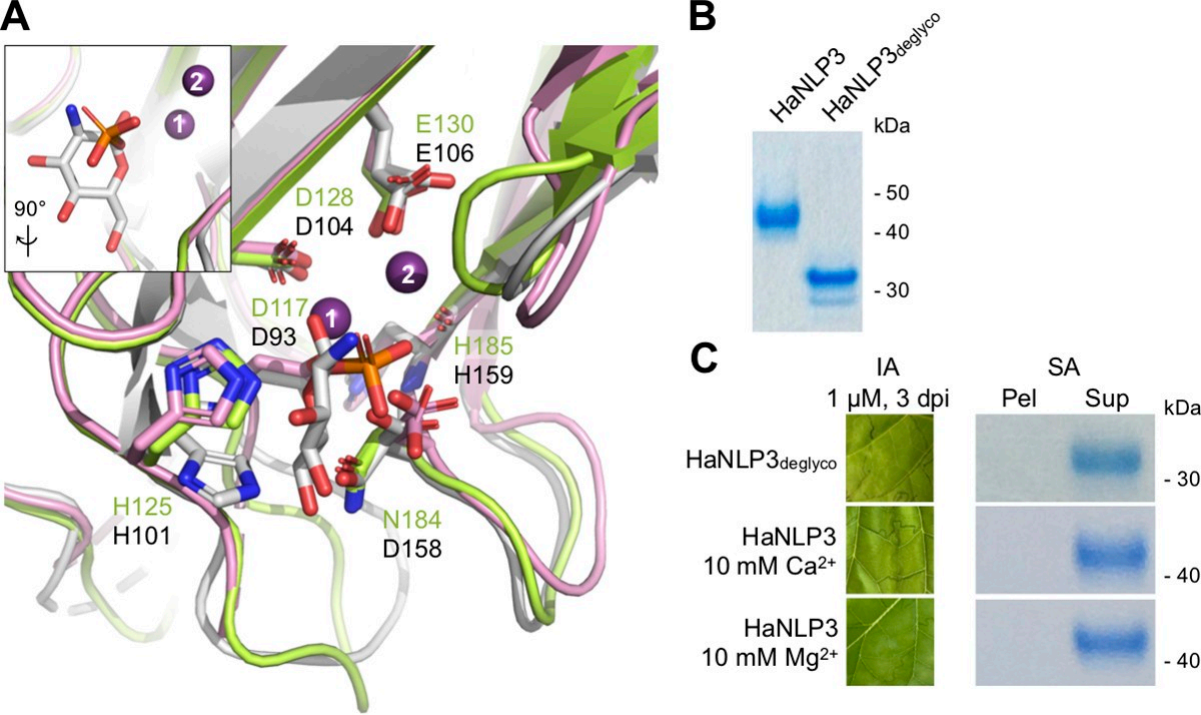
Detailed structural and functional analysis of non-toxic HaNLP3 and toxic NLP_Pya_. (A) Close-up view of glucosamine and Mg^2+^ binding sites in apo-NLP_Pya_ (white) and NLP_Pya_-glucosamine complex (pink) with respect to the HaNLP3 polypeptide chain (green). NLP_Pya_ amino acid residues involved in binding of glucosamine and ion coordination are highlighted and displayed as sticks (black text); all equivalent HaNLP3 residues are also shown (green text). Inset: Superposition of glucosamine and the phosphate ion in NLP_Pya_-glucosamine complex and HaNLP3, respectively, 90° relative to (A). Mg^2+^ ions are displayed as purple spheres: (1), position of Mg^2+^ in apo-NLP_Pya_; (2), position of Mg^2+^ in NLP_Pya_-glucosamine complex. Glucosamine (white) and phosphate ions (orange and red) are shown in sticks. (B) SDS-PAGE analysis of glycosylated (HaNLP3) and deglycosylated HaNLP3 (HaNLP3_deglyco_). (C) Infiltration assay (IA) detecting tobacco leaf necrosis after infiltration of HaNLP3_deglyco_ and HaNLP3 in the presence of Ca^2+^ or Mg^2+^, and binding to POPC:GIPCs multilamellar vesicles studied by liposome sedimentation assay (SA). Dpi, days post-infiltration; Pel, pellet; Sup, supernatant.

We recently demonstrated dislocation of the L3 loop of NLP_Pya_ upon hexose binding, which may mimic GIPC headgroup recruitment [11]. This results in the opening of the L2-L3 crevice and movement of a magnesium ion which is required for activity. All amino acid residues involved in Mg^2+^ binding in NLP_Pya_ [6] are preserved in HaNLP3 except for Asp158 (Fig 3A), which is also important for glucosamine binding in NLP_Pya_ [11] and is replaced by Asn184 in HaNLP3. However, no metal ion is present in the HaNLP3 crevice. HaNLP3 Asn184 is involved in phosphate positioning, which is however mainly contributed to by the side chains of His125 and His154 (Fig 3A). Interestingly, the phosphate ion bound to HaNLP3 is located close to the position of glucosamine in NLP_Pya_ (Fig 3A, inset).

Although HaNLP3 has a theoretical molecular weight of 28 kDa, it eluted from a size exclusion column with a retention time typical of a 45 kDa protein, suggesting glycosylation of the protein. The protein was deglycosylated (HaNLP3_deglyco_) with PNGase F, resulting in significantly faster SDS-PAGE migration (Fig 3B). Importantly, NLP_Pya_ was not glycosylated [6]. This difference may stem from the use of different expression systems. To exclude the possibility that HaNLP3 glycosylation blocks its toxic activity, HaNLP3_deglyco_ was tested for necrotic activity and for binding to GIPC-containing vesicles (Fig 3C). HaNLP3_deglyco_ was not able to induce necrosis or bind to vesicles, indicating glycosylation is not accountable for non-toxicity. Unlike NLP_Pya_, HaNLP3 does not possess a coordinated metal ion in its structure (Fig 2A). The Mg^2+^ ion is crucial for NLP_Pya_ toxic activity because of its indirect involvement in GIPC sugar headgroup binding [6, 11]. To test a requirement of metal ions for putative HaNLP3 toxin activity we also tested necrosis formation by HaNLP3 in the presence of divalent metal ions Ca^2+^ and Mg^2+^. Neither ion was able to promote HaNLP3 toxicity in tobacco leaves or binding to vesicles with embedded GIPCs (Fig 3C).

**Fig 4:**
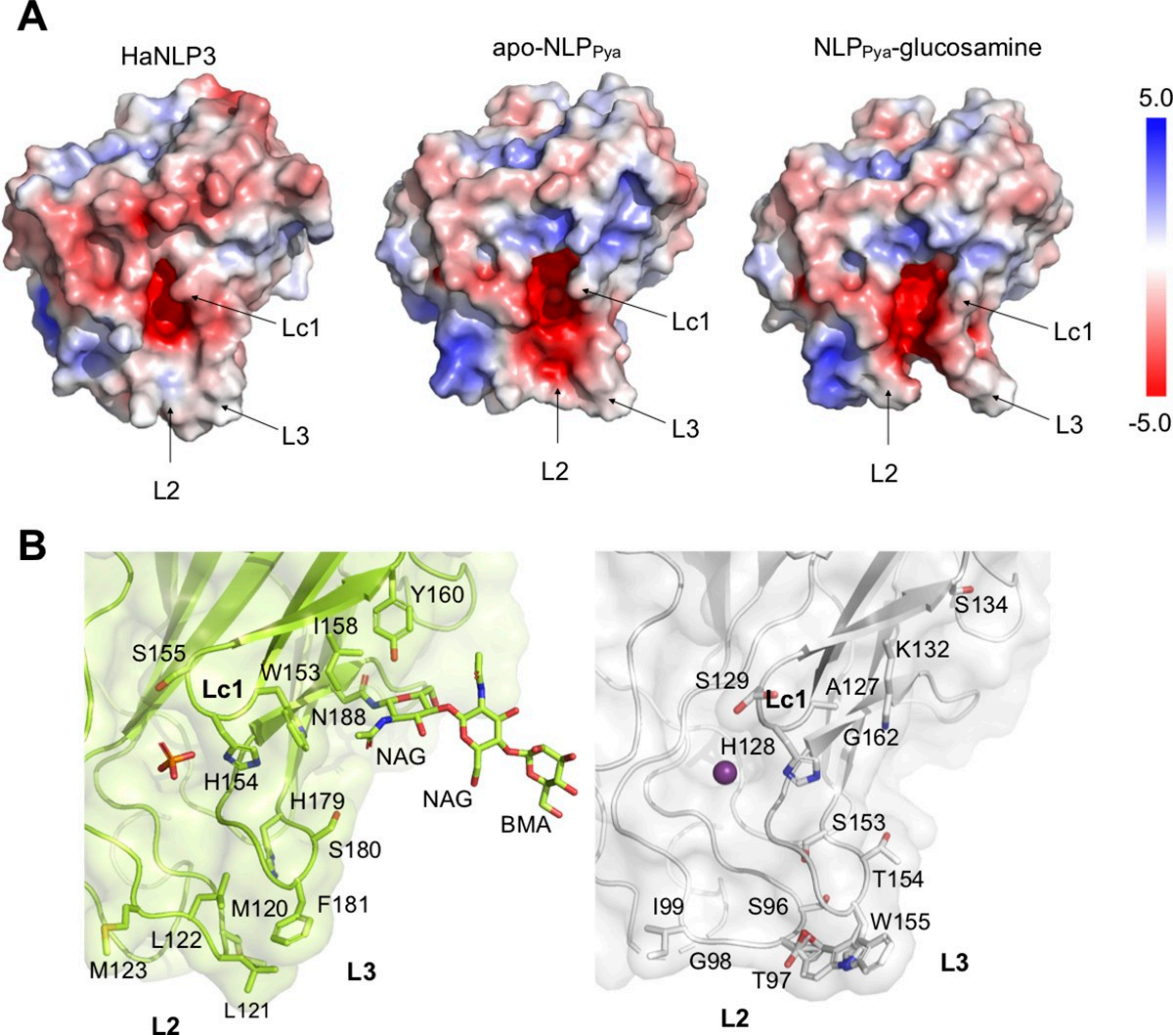
Comparison of protein surface features between non-toxic HaNLP3 and toxic NLP_Pya_. (A) Charges on the molecular surfaces of the HaNLP3, apo-NLP_Pya_ and NLP_Pya_-glucosamine complex. Red and blue represent negative charge and positive charge, respectively. All ligands are excluded for clarity. (B) Detailed comparison of Lc1, L2 and L3 loops. HaNLP3 amino acid residues (green) and equivalent NLP_Pya_ residues (white) are highlighted and shown in sticks. Phosphate and Mg^2+^ ion are displayed as orange/red sticks and purple sphere, respectively. NAG, N-acetylglucosamine; BMA, mannose.

Comparison of NLP_Pya_ protein surfaces in the apo- and hexose-bound states with HaNLP3 reveals that the crevice of HaNLP3 appears to be in a more closed state than the cavity of apo-NLP_Pya_ (Fig 4A). As calculated by CASTp [19], the volumes of the NLP_Pya_ and HaNLP3 crevices are 139.4 Å^3^ and 77.5 Å^3^, respectively. Comparison of the charges on the molecular surfaces of HaNLP3 and NLP_Pya_ before or after hexose binding reveals differences in the L2, L3 and Lc1 loops, which are remarkably more hydrophobic in the case of HaNLP3 than in NLP_Pya_ (Fig 4A). The negative charge protrudes from the center of the crevice towards the exterior of NLP_Pya_ protein, while in HaNLP3, the negative charge is limited to the center of the cavity.

One of the significant differences between the NLP_Pya_ and HaNLP3 structures is the conformation of a Lc1 loop, which affects the opening of the cavity and the overall positioning of hydrophobic amino acid residues in the vicinity of the Asn188 glycosylation site in HaNLP3 (Fig 4B). Detailed analysis uncovered a noteworthy amino acid residue substitution from NLP_Pya_ Ala127 to a bulkier Trp153 in HaNLP3, as well as the presence of hydrophobic Ile158 and Tyr160 in HaNLP3 instead of Lys132 and Ser134 in NLP_Pya_ (Fig 4B, S2 Fig). Furthermore, amino acid sequences of the loops L2 and L3 are distinct in toxic and non-toxic NLP representatives (Fig 4B, S2 Fig). L2 loop amino acid composition differs between HaNLP3’s Met120-Leu121-Leu122-Met123 and NLP_Pya_’s Ser96-Thr97-Gly98-Ile99 (Fig 4B, S2 Fig). Trp155 in loop L3 of NLP_Pya_ was shown to be crucial for necrosis, probably due to initial interaction with the plasma membrane [11]. In the case of HaNLP3, the sequence His179-Ser180-Phe181 is present instead of Ser153-Thr154-Trp155 (Fig 4B, S2 Fig). This hydrophobic environment may contribute to HaNLP3 conformational rigidity along with its inability to recruit the GIPC sugar headgroup and consequently interact with the lipid membrane.

### Mutational analysis reveals important functional motifs

**Fig 5:**
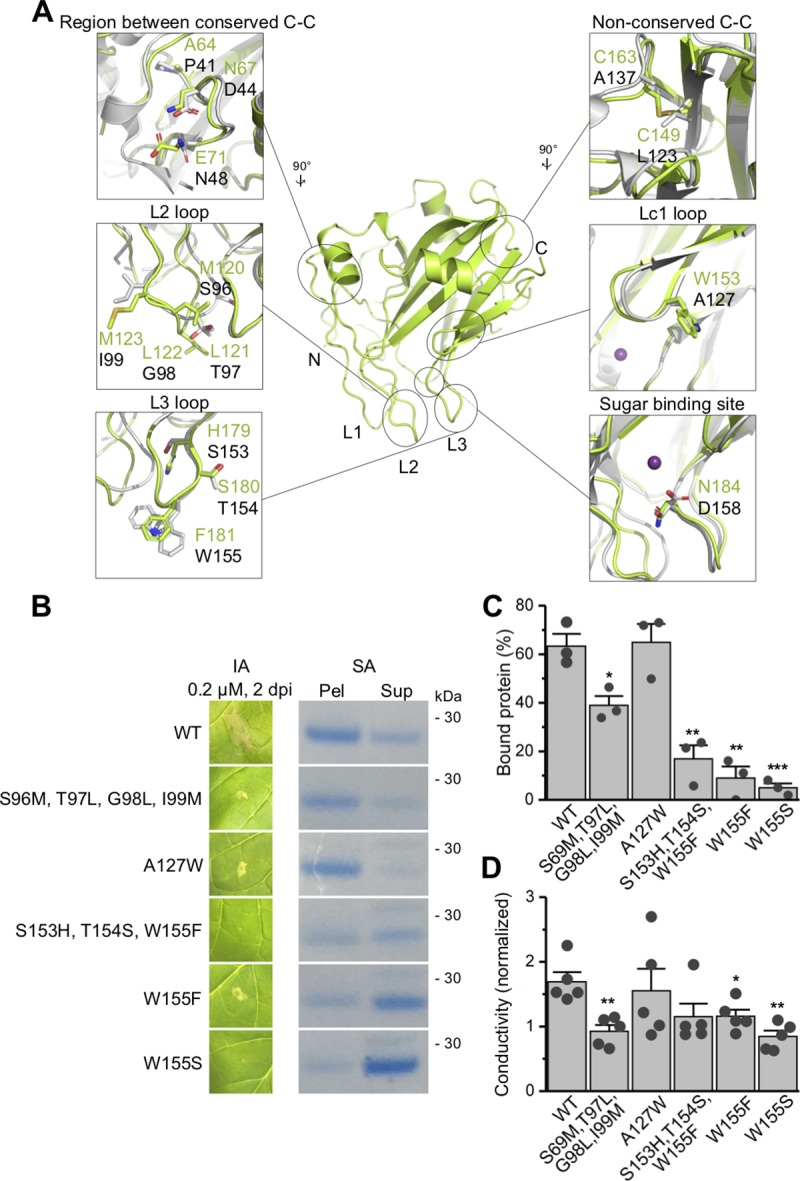
Mutational analysis of putative functional motifs in NLPs. (A) Detailed overview of sequence-based structural differences between non-toxic HaNLP3 (green) and toxic NLP_Pya_ (white) in the region between conserved cysteines and loops Lc1, L2 and L3. Proteins differ in the presence of non-conserved disulfide and sugar binding sites, as well. Selected HaNLP3 amino acid residues (green text) and equivalent NLP_Pya_ (black text) residues are highlighted and shown in sticks. (B) Tobacco leaf necrosis upon protein infiltration assay (IA) and binding of NLP_Pya_ proteins to POPC:GIPCs multilamellar vesicles, monitored by liposome sedimentation assay (SA). Dpi, days post-infiltration; Pel, pellet; Sup, supernatant. (C) Protein bands from liposome sedimentation assay from (B) were statistically analysed after densitometric quantification using image analyser system. Values are means ± SEM (n = 3), ***P<0.001 **P<0.01, *P<0.05 vs. control (the wild-type protein, WT). (D) Quantification of cell death by means of ion leakage experiment measurement. Values are normalized to the background value of the deionized distilled water and are shown as means ± SEM (n = 5), ***P<0.001 **P<0.01, *P<0.05 vs. control (WT).

Close examination of the HaNLP3 structure in comparison with the structure of NLP_Pya_ [6] and other toxic NLPs revealed a set of unique structural features worth further investigation. For example, it has been shown that swapping the region between oomycete type 1 NLP conserved cysteine residues with that of toxic PsojNIP from *Phythophtora sojae* was crucial for gain of NLP necrotic activity in HaNLP3 [15]. A multiple amino acid sequence alignment of HaNLP3 protein with several toxic NLP representatives in the region between these two cysteines pinpointed a few possible amino acid residues that could affect NLP toxicity: Ala64, Asn67 and Glu71 in non-toxic HaNLP3 and corresponding Pro41, Asp44 and Asn48 in toxic NLP_Pya_ (Fig 5A, S2 Fig). Furthermore, HaNLP3 contains a second disulfide bond unlike NLP_Pya_ (Fig 5A, S2 Fig), which could play a part in HaNLP3 rigidity when interacting with the membrane.

In order to examine the significance of these sequence differences, selected equivalent HaNLP3 amino acid residues were introduced individually or in combination via mutagenesis into corresponding positions of NLP_Pya_ sequence. The resulting mutant proteins were analysed for toxicity using the tobacco leaf infiltration assay (Fig 5A and 5B, S3A Fig, S1 Table). To exclude the possibility that effects of mutations on necrotic activity were a consequence of protein instability, CD measurements and differential scanning fluorimetry assays were performed. All mutants retained their secondary structure according to CD spectra (S3B Fig). However, several of them exhibited lower thermal stability compared to the wild-type NLP_Pya_ (S3C and S3D Fig, S2 Table). As expected, proteins with low thermal stability (S3D Fig) displayed somewhat reduced necrotic activity as shown by an infiltration using tobacco leaves (S3A Fig). Thus these mutations were excluded from analysis. In general, proteins with moderate or high stability showed no significant effect on toxic activity except for the NLP_Pya_^A127W^ (Lc1 loop), NLP_Pya_^S96M, T97L, G98L, I99M^ (L2 loop), NLP_Pya_^S153H, T154S, W155F^, NLP_Pya_^W155F^, and NLP_Pya_^W155S^ (all L3 loop) mutants. Liposome sedimentation assays employing POPC:GIPCs multilamellar vesicles confirmed compromised membrane binding ability of NLP_Pya_^W155F^, NLP_Pya_^S153H, T154S, W155F^, NLP_Pya_^S96M, T97L, G98L, I97M^, NLP_Pya_^W155S^, but not of NLP_Pya_^A127W^ (Fig 5B and 5C). We also performed ion leakage assays and observed similar effects of these mutations on the ability to cause cell death (Fig 5D).

To determine whether slightly less toxic NLP_Pya_^P41A, D44N, N48E^ (S3 Fig) displays any structural divergence from the wild-type protein, we determined its crystal structure (S4A Fig, Table 1). Except from the triple mutation, the overall structure strongly resembles the wild-type NLP_Pya_ structure with C_α_ RMSD value of 0.3 Å and 0.5 Å for chain A and chain B, respectively (calculated by DALI [18]) (S4B Fig). As shown in our previous work, sugar binding causes the Mg^2+^ ion to shift towards interior of the NLP_Pya_ so it becomes coordinated by a different amino acid set than in apo-NLP_Pya_ [11] (S4C Fig). However, positions of Mg^2+^ ions in other polypeptide chains in the asymmetric unit that did not bind the hexose, were in between the complex-form and apo-form of NLP_Pya_ [11] (S4C Fig). Interestingly, in the NLP_Pya_^P41A, D44N, N48E^ we observed another variation of the Mg^2+^ ion octahedral coordination. Here, the Mg^2+^ ion is directly coordinated by Asp104 and Glu106 and indirectly by Asp93 and His159 via four water molecules (S4C Fig). This further highlights that Mg^2+^ coordination can adjust to the spatial arrangement of different NLP_Pya_ scaffolds.

In summary, these results indicate an important functional role of L2 and L3 loops, and, to some extent, also of loop Lc1, for effective embracement of the GIPC headgroup. Furthermore, it is evident how a small allosteric change, for instance a triple mutation in NLP_Pya_^P41A, D44N, N48E^, affects characteristic structural features of NLP_Pya_.

### NLP proteins differ in functional plasticity of the GIPC headgroup binding cavity

**Fig 6:**
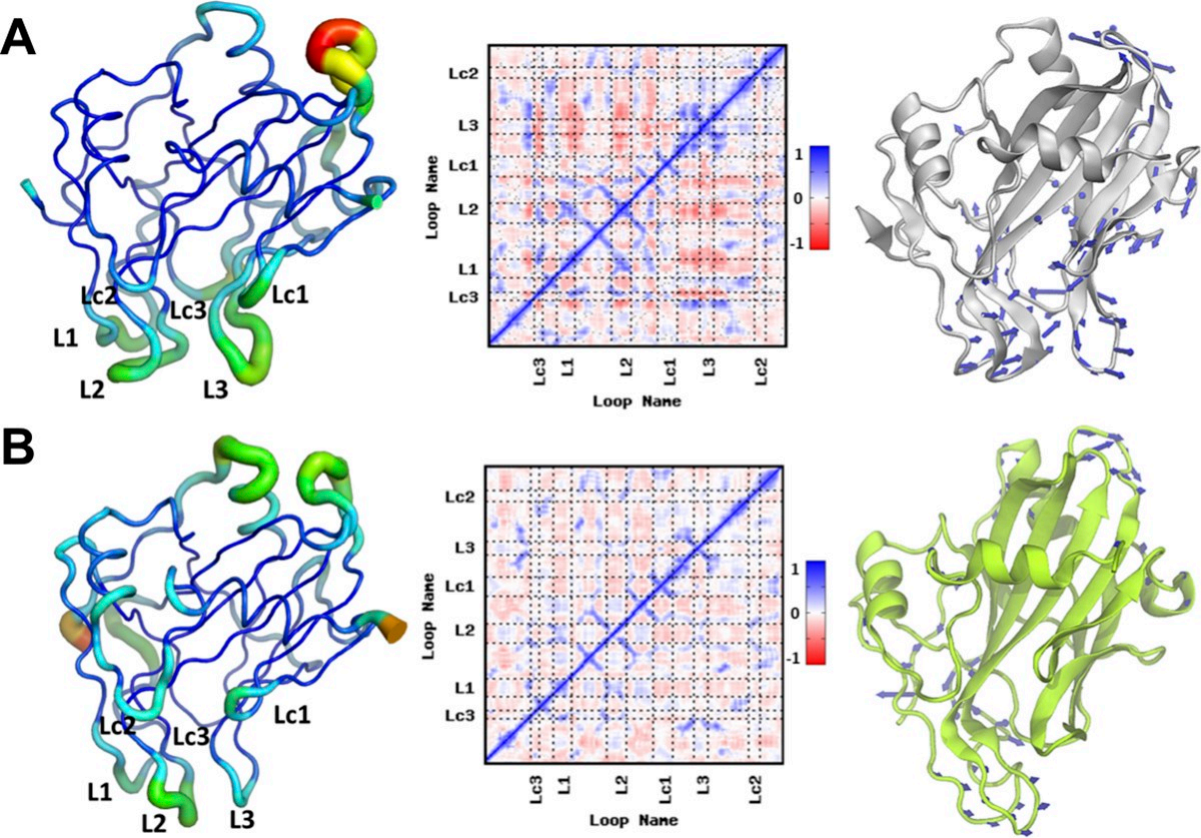
Dynamical properties of NLP_Pya_ and HaNLP3. The first column depicts graphical representations of root mean square fluctuation (RMSF) per residue in (A) NLP_Pya_ and (B) HaNLP3 projected on the most representative cluster of the protein (shown as tube) as extracted from the MD trajectories. Colors from red to blue along with a decreasing size of the tube indicate a progressive decrease of the RMSF. In the second column, we report the cross-correlation matrices. Correlation scores ranges from +1 (red) to -1 (blue). A positive sign of the map (blue color) indicates that residues move in a correlated manner (lockstep), otherwise, a negative value (red color) points to an anti-correlated motion between residues (opposite directions). White color shows that residue displacements are independent from each other. Third column shows the essential dynamics of the proteins. These are represented as white and green cartoons for (A) NLP_Pya_ and (B) HaNLP3, respectively. Blue arrows indicate the direction and the amplitude of the motion.

Upon discovering the important functional role of the loops at the bottom of the molecule and differential surface properties, we were interested in gaining perspective on how NLP flexibility affects its ability to bind GIPCs. In order to identify the key dynamic traits at the basis of toxic versus non-toxic activity of the NLP_Pya_ and HaNLP3, we performed cumulative multi-μs-long molecular dynamics (MD) simulations in explicit solvent. These simulations revealed that the two proteins display a remarkably different conformational flexibility (measured as root mean square fluctuation (RMSF) per residue) of the L1, L2 and L3 and Lc1 loops lining the GIPC headgroup binding cavity, with the L2 and L3 being significantly more flexible in NLP_Pya_ (Fig 6). The restricted conformational plasticity of the L3 loop in HaNLP3, compared to NLP_Pya_, is caused by the formation of persistent hydrogen bonds (H-bonds) connecting L3 with Lc1 and Lc3 (S3 and S4 Tables). Notably, the NLP_Pya_^P41A, D44N, N48E^ mutant exhibits a limited flexibility of L1, L2, and Lc3 as compared to the wild-type protein (S5 Fig), in line with its reduced necrotic activity. The mutations induce the loss of a persistent H-bond (between side chains of Asp44-Asn48, S5 Table), which in NLP_Pya_ firmly links Lc3 to L1, affecting their flexibility.

To further assess the functional role of the loops lining the GIPC-binding cavity, we inspected the presence of possible correlated motions among different structural elements of the proteins by computing a correlation analysis based on the Pearson coefficients. The cross-correlation matrix of NLP_Pya_ (Fig 6) indicates a dynamical coupling among the loops. Namely, L1, L2, Lc1 and Lc3 positively correlate with each other, moving in lockstep, while all negatively-correlate with L3, which moves in an opposite direction. This correlation is not predicted in HaNLP3, consistent with the observed higher rigidity of the loops (Fig 6). The existence of a finely tuned collective functional dynamics is further corroborated by the disappearance of the positive correlation in NLP_Pya_^P41A, D44N, N48E^. The loss of H-bonds linking Lc3 with L1 (S5 Table) induced by these mutations not only reduces the thermal stability of NLP_Pya_^P41A, D44N, N48E^, but also affects the protein internal movements.

Next, in order to characterize how the observed differences of the cross-correlation matrices translate into distinct large-scale collective motion of proteins, we have also captured the essential dynamics of the systems by performing Principal Component Analysis (PCA) of the NLP_Pya_ and HaNLP3 MD trajectories. The principal mode of motion (Principal Component 1, PC1) of NLP_Pya_, pictured by vectors indicating the direction and the amplitude of the motion (Fig 6), underpins an opening/closing of loops at the entrance of the lipid binding cavity. This motion is most likely functionally linked with the loading and embracing the GIPC headgroup (S1 Movie). The opening and closing of the binding cavity almost vanishes in HaNLP3 (S2 Movie), and it is markedly dampened in NLP_Pya_^P41A, D44N, N48E^.

As an additional check, we analyzed the functional plasticity of the toxic NLP protein MpNEP2 [7]. A graphical plot of the RMSF pinpoints a different type of flexibility, which involves mainly Lc1, Lc2 and to a lesser extent L2 and L3 (S6 Fig). Nevertheless, the cross-correlation matrix exhibits remarkable similarities to NLP_Pya_ in terms of the coupled motion of different loops. As a result, the essential dynamics of MpNEP2 displays an opening/closing of the sugar headgroup-binding cavity mostly ascribable to Lc1 and Lc2 (S6 Fig). Altogether, these results suggest that the protein flexibility and the movement of the loops affecting opening/closing of the lipid-binding site is critical for protein toxicity.

### Function is partially recovered by incorporation of plastic regions into HaNLP3 scaffold

**Fig 7:**
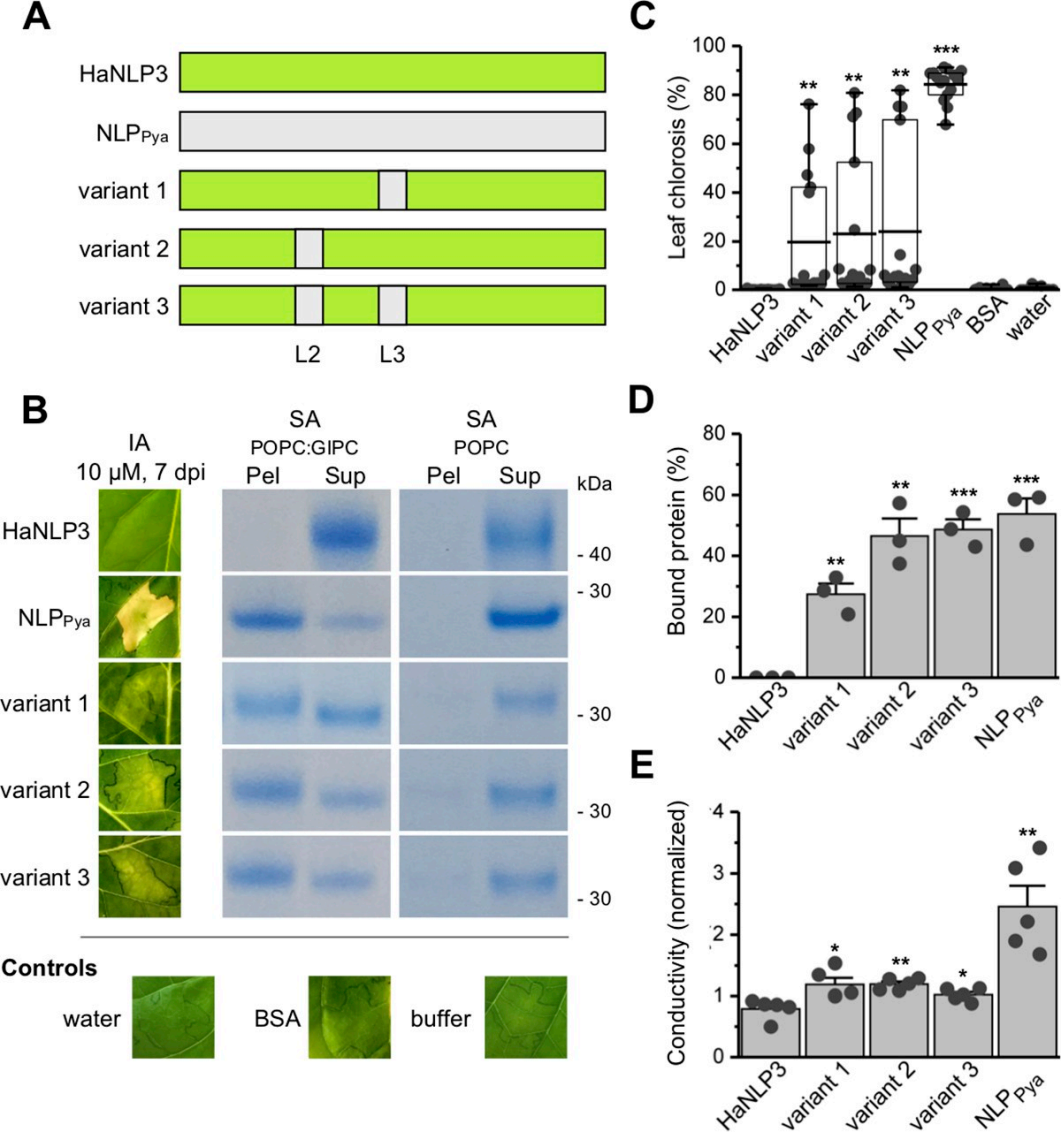
Necrosis-inducing ability of HaNLP3 and variants 1, 2 and 3. (A) Schematic representation of the swapped regions from NLP_Pya_ to HaNLP3 scaffold. (B) Tobacco leaf infiltration assay (IA) and binding of HaNLP3, NLP_Pya_ and variants 1, 2 and 3 to POPC:GIPCs multilamellar vesicles monitored by means of liposome sedimentation (SA). Dpi, days post-infiltration; Pel, pellet; Sup, supernatant; Buffer, 20 mM Tris pH 7.5 and 150 mM NaCl. (C) Statistical analysis of leaf chlorosis (5–7 dpi). Values are means ± SEM (n = 15), ***P<0.001 **P<0.01, *P<0.05 vs. control (deionized distilled water). (D) Statistical analysis of protein bands from liposome sedimentation assay from (b) using image analyser system. Values are means ± SEM (n = 3), ***P<0.001 **P<0.01, *P<0.05 vs. control (HaNLP3). (E) Quantification of cell death by means of ion leakage experiment measurement. Values are normalized to the background value of the deionized distilled water and are shown as means ± SEM (n = 5), ***P<0.001 **P<0.01, *P<0.05 vs. control (HaNLP3).

In order to test the functional role of Lc1, L2 and L3 loops, we performed MD simulations of three HaNLP3 variants, into which Lc1 and L3 from NLP_Pya_ (variant 1), Lc1 and L2 from NLP_Pya_ (variant 2) and Lc1, L2 and L3 loops from NLP_Pya_ (variant 3), respectively, were introduced (Fig 7A). MD simulations revealed that the introduction of new amino acid sequences in HaNLP3 variant 1 caused an increase of mobility of the L3 and Lc1 with respect to that observed in wild-type HaNLP3 (S7A Fig, S5 Table). However, this effect was not so obvious when variants 2 or 3 were tested (S7 Fig). In addition, dynamical lockstep movement coupling among L1, L2, Lc1 and Lc3 and the opening/closing of the lipid-binding cavity, yielding from PC1, is partially recovered in HaNLP3 variant 1, while the motion of the loops remained uncorrelated in variants 2 and 3 (S7 Fig).

To experimentally confirm MD results, we produced HaNLP3 variants 1, 2 and 3 and tested them for activity (Fig 7A, S6 Table). HaNLP3 and NLP_Pya_ share 45% identical amino acid residues. Apart from the ones that are changed in the region, responsible for lipid headgroup binding, there are also other numerous amino acid changes that may affect the subsequent steps in the cytolytic process on plant cell membranes. We anticipated that the restoration of the toxic phenotype would not be possible to a full extent. Therefore, we have employed higher protein concentrations and longer time frames in leaf infiltration assays (S8 Fig). In contrast to HaNLP3, deionized distilled water and BSA, all variants (1, 2 and 3) caused leaf necrosis, but to a lesser extent than NLP_Pya_ (Fig 7B and 7C). Although the necrosis of HaNLP3 variants was not as clear as in the case of NLP_Pya_ and significantly higher concentrations were needed, weak leaf damage manifested as chlorosis in a concentration dependent manner (S8 Fig). Toxic HaNLP3 variants also bound to liposomes in a GIPC-dependent manner similar to NLP_Pya_ (Fig 7B and 7D). Moreover, ion leakage experiments confirmed these results for variant 1, as we were able to detect an increase in toxicity in comparison to HaNLP3. To a lesser degree, this was also observed for variants 2 and 3 (Fig 7E). Altogether, these experiments confirm an essential role of plastic loops in NLP_Pya_ together with the amino acid content of the L2/L3 crevice as crucial for GIPC binding and toxicity.

## Discussion

Plant pathogens represent a substantial threat to global food security as plant diseases cause reduction of agronomic productivity leading to enormous economic loss [20, 21]. Plants co-evolved with plant pathogens, leading to the development of a multilayered surveillance system to defend against and fight microbe invasion [22–25]. NLP proteins are produced by various phytopathogenic microorganisms and can stimulate plant innate immunity as well as cause cell death in eudicot plants [1, 3, 4, 6, 26].

Non-toxic NLPs have been identified in multiple (hemi)biotrophic pathogens, suggesting functional diversification within this microbial protein superfamily [5, 15, 27]. Biotrophic infection strategies do not rely on host plant cell death, and are considered to have evolved more recently than necrotrophic or saprophytic forms of microbial parasitism. Unlike toxic NLPs, which tend to be expressed late in infection, the non-toxic counterparts in HaNLPs are expressed at early stages of infection [15]. In addition, some non-toxic NLP-encoding genes have been shown to be under positive selection [27]. Altogether, these observations have led to the hypothesis that non-toxic NLPs have evolved during expansion of and neofunctionalization within the NLP family to adopt new, as yet unknown, functions unrelated to NLP cytotoxicity [5, 27]. At present, we and others are not able yet to demonstrate whether non-cytolytic NLPs contribute to microbial infection, and what might be their biochemical activity. This is mainly because of the functional redundancy of non-cytolytic NLP-encoding genes and/or genetic intractability of the microbes that produce these proteins, i.e. *H*. *arabidopsidis*. For example, *H*. *arabidopsidis*, an obligate biotroph, produces HaNLP3 in addition to 9 other non-cytolytic NLPs [15]. Likewise, *Phytophthora sojae*, a hemibiotroph, produces 8 cytolytic NLPs in addition to 12 non-cytolytic NLPs [27]. Together, with significant sequence conservation among cytolytic and non-cytolytic NLPs, this may illustrate the methodological challenges in demonstrating involvement of these proteins in microbial virulence by means of reverse genetics. The work presented in this manuscript thus importantly contributes to our understanding of NLPs diversification in relation to the first step of the intoxication process, binding to lipid membrane of the target cell.

This study provides the first functional and structural analysis of non-toxic oomycete type 1 HaNLP3, produced by *H*. *arabidopsidis* in the early stages of *Arabidopsis thaliana* infection [15]. Due to its high sequence similarity to toxic NLP_Pya_, HaNLP3 represents an excellent model protein to investigate differences between toxic and non-toxic NLP variants. Recently, we proposed that the principal functional feature of toxic NLPs is their association with the GIPC headgroup, followed by disruption of plasma membrane integrity which leads to cell death in eudicot plants [11]. Here we disclose that the functional difference between toxic NLP_Pya_ and non-toxic HaNLP3 protein is in GIPC headgroup recognition. In contrast to NLP_Pya,_ the HaNLP3 protein does not bind to GIPCs alone or GIPCs embedded in liposomes, consistent with its inability to cause necrosis of tobacco leaves.

We have solved the HaNLP3 crystal structure to explore the molecular mechanism of HaNLP3 non-toxicity. While the overall fold between HaNLP3 and toxic NLP_Pya_ is highly conserved, there are important structural differences between the two types of NLPs. For example, HaNLP3 is glycosylated, contains an additional cysteine bond, and lacks a bivalent metal ion. There is no information available about HaNLP3 glycosylation patterns when produced in its native host *H*. *arabidopsidis*. Due to expression of recombinant HaNLP3 in *P*. *pastoris* we subsequently performed protein deglycosylation assays to exclude possible effects of glycosylation on toxicity. We showed that deglycosylation or preincubation with metal ions did not affect HaNLP3 non-toxicity; nor did the introduction of an analogous disulfide bond in NLP_Pya_ change its toxicity. Importantly, comparing the surfaces of HaNLP3 and NLP_Pya_ proteins showed a volume disparity in the Mg^2+^ and hexose binding cavity. In HaNLP3, the cavity volume is significantly smaller than in NLP_Pya_. Structural differences based on variation at the level of amino acid sequences between HaNLP3 and NLP_Pya_ in the Lc1, L2 and L3 loops appear to be critical for the gain/loss of necrotic activity. Several amino acids that are presumably important for toxicity have already been identified based on toxic and non-toxic NLP amino acid sequence comparisons; yet the effect of amino acid substitutions on necrosis was tested mostly by *Agrobacterium*-mediated plant transformations [28–33]. In this study, we performed biophysical characterization of recombinant NLPs, analysed their thermal stability and characterized their dynamic properties. This analysis highlighted the importance of plasticity and dynamical coupling of the loops surrounding the binding cavity as key factors regulating protein stability and toxicity. Swapping these regions from HaNLP3 to NLP_Pya_ resulted in reduced GIPC binding ability. Furthermore, the NLP_Pya_^P41A, D44N, N48E^ mutant, functionally and structurally characterized in this work, pinpoints another important structural element to regulate stability and functional dynamics of the protein. This underlines how additional switch elements affecting Mg^2+^ and GIPC headgroup binding may be spread across distinct protein regions.

Varied amino acid content and positioning of the Lc1, L2 and L3 loops around the GIPC headgroup binding cavity may affect local and overall dynamic properties of HaNLP3 and NLP_Pya_. Indeed, the essential dynamics of NLP_Pya_ revealed a collective motion of the Lc1, L1, L2 and L3 loops, which may enable opening and closing of the lipid-binding cavity. Such a dynamic arrangement is most likely a prerequisite for the capability of NLP_Pya_ to recognize the GIPC sugar headgroup and to associate with eudicot plant membranes. Remarkably, the toxic MpNEP2 [7] protein shares the same type of functional movement. This provides compelling evidence for a general mechanism of toxic NLPs embedded into the highly conserved structural scaffold, irrespective of the exact primary structure. Conversely, HaNLP3 is typified by a limited conformational plasticity of these loops. Introduction of the flexible Lc1, L2 and L3 loops of NLP_Pya_ onto the non-toxic HaNLP3 scaffold resulted in slightly increased toxicity upon infiltration into tobacco leaves and enhanced binding capacity towards multilamellar vesicles containing GIPCs compared to wild-type HaNLP3. Consistent with these data, MD simulations of these mutants partially recover the functional plasticity of the loops observed in NLP_Pya_, clearly pointing to L2, L3/Lc1 as key switch elements of toxicity. Interestingly, HaNLP3 variants are almost equivalent to NLP_Pya_ in terms of binding to multilamellar vesicles, however, they differ significantly in terms of their toxic effects as shown by infiltration assays and conductivity measurements (Fig 7). NLPs-mediated pore formation and host cell death is likely to be a multi-step process, in which individual parts of the toxin contribute differently to distinct stages in pore formation. In summary, we established that lack of cytotoxicity of HaNLP3 is the result of key amino acid differences spread across the molecule. Small differences in primary structure collectively contribute to the absence of toxicity. These differences include critical amino acids of the loops around the binding cavity which affect either the conformational plasticity (Lc1, L2 and L3 loops) of the binding cavity and/or its membrane anchoring potential (residue Trp155 and residues nearby), as well as amino acids at more distal sites (Pro41, Asp44, Asn48). Our data suggest that evolution of non-toxic NLP proteins has likely been driven by the accumulation of mutations affecting conformational plasticity of the GIPC binding site and tryptophan anchoring. Ultimately, toxic activity was abolished and resulted in neofunctionalization of a former cytotoxin. We clearly show that key switch elements regulating non-toxic and toxic activity are hidden within distinct parts of the NLP proteins and that the resulting lack of toxicity is controlled by sets of mutations at key sites, which primarily affect the GIPC headgroup-binding site.

## Materials and methods

### Expression and purification of HaNLP3, NLP_Pya_ and their mutants

The HaNLP3 protein was expressed and purified as described previously [15] with the following modifications. The supernatant of *Pichia pastoris* culture medium was treated with 60% (w/v) ammonium sulfate and centrifuged at 24,523 *g* and 4°C for 20 min. The precipitate was resuspended in deionized distilled water and loaded onto Q Sepharose FF ion-exchange column, equilibrated in 20 mM Tris buffer pH 9. Protein was eluted with a 0 to 0.5 M NaCl gradient in 20 mM Tris buffer pH 9. Fractions containing HaNLP3 protein were pooled, concentrated and subjected to a HiLoad 26/600 Superdex 75 column equilibrated in 20 mM Tris buffer pH 7.5, 150 mM NaCl and 5% (v/v) glycerol. Protein-containing fractions were pooled and dialyzed against deionized distilled water. The protein was concentrated and stored at -80°C. All the purification steps were followed by SDS-PAGE analysis. HaNLP3 variants were expressed in *Escherichia coli* BL21(DE3) in TB medium. Cells were grown at 37°C until absorbance at 600 nm reached value of 1, followed by an overnight induction with 0.5 mM isopropyl 1-thio-β-D-galactopyranoside at 20°C. Cells were then harvested by centrifugation at 4°C and 5,647 *g* for 10 min and resuspended in lysis buffer (50 mM Tris pH 7.5, 250 mM NaCl and 10% (v/v) glycerol). Cell debris was removed by centrifugation at 50,000 *g*, 4°C for 1 h after sonication. The supernatant was filtered through 0.22 μm filter and loaded to a Ni-NTA column (Qiagen), equilibrated with 50 mM Tris pH 7.5, 250 mM NaCl and 10 mM imidazole. The bound fraction was washed with 50 mM Tris pH 7.5, 250 mM NaCl and 20 mM imidazole and eluted within the gradient of imidazole from 20 to 500 mM. Fractions that contained HaNLP3 variants were pooled and dialysed against 20 mM Tris pH 7.5 and 150 mM NaCl, concentrated and stored at -80°C until used. *E*. *coli* production of NLP_Pya_ and its mutants was performed as reported in Lenarčič 2017 [11].

### Deglycosylation

Deglycosylated HaNLP3 protein was prepared using recombinant N-glycosidase F enzyme according to the Roche kit instructions (3U enzyme/10 μg protein).

### Circular dichroism

Far UV CD spectra of NLP_Pya_ and its mutants were recorded on a Chirascan spectrometer (Applied Photophysics) using 0.08 mg/ml protein in 20 mM MES pH 5.8 in a 0.1-cm path length quartz cuvette. Spectra were measured in a 200–250 nm wavelength range with a step size of 0.5 nm and integration time of 0.5 s at 20°C. Each spectrum represents the average of five scans. Spectra were processed, baseline corrected, smoothed and converted with the Chirascan software. Spectral units were expressed as the mean molar ellipticity per residue.

### Thermal stability assay

The thermal stability of NLP_Pya_ and its mutants was measured by differential scanning fluorimetry (thermofluor) assay using 2x SYPRO Orange dye (Thermo Fisher Scientific). Protein concentration was 0.1 mg/ml. Thermal stability of proteins was tested in 20 mM MES pH 5.8 and 150 mM NaCl. Samples were subjected to a temperature gradient from 25°C to 85°C at a 1°C/min step. Temperature melting profiles were acquired with LightCycler 480 (Roche). Melting temperatures T_m_ were determined by using Boltzmann function in Origin 8.1 (Origin Lab). Each T_m_ value represents the average of six repeats from three independent experiments.

### Sequence alignment

The amino acid sequences of non-toxic HaNLP3 and toxic NLPs without signal peptide sequences were aligned using Clustal Omega [34].

### Infiltration assay

The infiltration assay was performed on tobacco plants. 50 or 100 μl of proteins (diluted in deionized distilled water) with a concentration 0.2 μM, 1 μM or 10 μM were infiltrated into tobacco leaves using a syringe without a needle. Lesion formations were examined 3–7 days post-infiltration. To test the effect of metal ion presence on HaNLP3 toxicity, 1 μM HaNLP3 was preincubated with 10 mM CaCl_2_ or 10 mM MgCl_2_ for 15 min. The level of leaf chlorosis/necrosis induced by HaNLP3 variants was determined using ImageJ software by calculating the ratio of the chlorotic area to the whole infiltrated area, both measured in squared pixels. Each value represents the average of fifteen infiltrations.

### Ion leakage

Leaves of tobacco were infiltrated with 0.5 μM (NLP_Pya_ mutants) and 10 μM (HaNLP3 variants) proteins. After 10 min incubation, 8 leaf disks were punched out (Ø 6 mm) and transferred to 10 ml of deionized distilled water. After 30 min of shaking at 50 rpm, leaf disks were transferred to 4 ml of fresh deionized distilled water. Conductivity was measured after 2 h using 712 Conductometer (Metrohm). All values were normalized to mock treated samples (leaves infiltrated with deionized distilled water). Each value represents the average of five replicates.

### Extraction and purification of GIPCs

GIPCs were prepared as previously described [11, 35]. Briefly, tobacco leaves were blended with cold 0.1 N aqueous acetic acid and filtered through miracloth. The slurry was then extracted with hot 70% ethanol/0.1 N HCl. The pellet was washed with cold acetone and diethyl ether to yield a whitish precipitate. This GIPC extract was dissolved in tetrahydrofuran (THF):methanol:water (4:4:1, v:v:v) containing 0.1% formic acid, dried and submitted to a butan-1-ol:water (1:1, v:v) phase partition. The upper, GIPC-containing butanol phase was dried and the residue was dissolved in THF:methanol:water (4:4:1, v:v:v) containing 0.1% formic acid. GIPCs were characterised by MALDI-MS [35] and their mass was estimated from dry weight.

### Liposome preparation

GIPC-containing liposomes were prepared by mixing POPC (Avanti Polar Lipids, Alabaster, AL) and GIPCs at the appropriate mass ratios, i.e. 1:3, 1:5 or 1:6. Different mass ratios of POPC *vs*. GIPC correspond to three independent GIPC isolation batches and were adjusted to ensure comparable NLP_Pya_ binding. Multillamelar vesicles (MLVs) were prepared by hydration of POPC or POPC:GIPCs thin lipid films in warm 20 mM MES, 150 mM NaCl, pH 5.8 and vortexing of the lipid suspensions in the presence of glass beads. Small unilamellar vesicles (SUVs) were obtained by sonication of MLVs on ice for a total of 30 min, with 10 s on-off cycles at 40% amplitude using a Vibracell Ultrasonic Disintegrator VCX 750 (Sonics and Materials, Newtown, USA).

### Liposome sedimentation assay

Liposome sedimentation assay was performed as previously described [11]. Briefly, NLP_Pya_ and HaNLP3 proteins and their mutants (0.06 mg/ml) were incubated with multilamellar vesicles (4.5 mg/ml) composed of POPC or POPC:GIPCs in 20 mM MES, 150 mM NaCl, pH 5.8, for 30 min and 600 rpm at room temperature. To test the effect of metal ion presence on HaNLP3—GIPC interaction, 10 mM CaCl_2_ or MgCl_2_ were added to the buffer. The mixture was centrifuged, and the liposomes were washed twice with the same buffer and subjected to SDS-PAGE, followed by Coomassie Brilliant Blue staining. Protein bands were statistically analyzed after densitometric quantification using image analyser system (ImageJ 1.29×, N.I.H., USA). Each value represents the average of three independent experiments.

### Surface plasmon resonance (SPR)

Binding analysis was conducted using Biacore T100 (GE Healthcare) docked with Series S sensor chip L1 (GE Healthcare). The system was first primed with SPR running buffer (20 mM MES, 150 mM NaCl, pH 5.8). After regenerating the chip, POPC SUVs (200 × diluted in running buffer) were injected over the first flow cell while POPC:GIPCs SUVs were captured on the second flow cell (10 min injection at 2 μl/min). The surface was then conditioned with a 120 s injection of 0.1 mg/ml BSA at 10 μl/min. The proteins were injected in a single cycle mode in concentrations 62.5, 125, 250, 500 and 1000 nM.

The binding of lipids to proteins was performed using Series S sensor chip CM5. Firstly, NLP_Pya_ and HaNLP3 were immobilized covalently via amine groups to the separate flow cells on the sensor surface with final immobilization level of 3800 RU for NLP_Pya_ and 4500 RU for HaNLP3. The binding of 10 μM lipids was monitored for 180 s followed by dissociation time of 180 s using SPR running buffer at a 10 μl/min flow rate. The removal of bound lipids was achieved using two short (24 s, 30 s) pulses of 0.07% SDS.

### Crystallization and structure determination

HaNLP3 (20 mg/ml) and NLP_Pya_^P41A, D44N, N48E^ (25 mg/ml) proteins were crystallized in the presence of 0.2 M di-ammonium hydrogen phosphate and 20% (w/v) PEG 3350 by sitting drop vapor diffusion, and 22% (w/v) PEG 4000, 0.3 M MgCl_2_, 0.1 M Tris pH 9, 10% (v/v) glycerol and 5% (v/v) methanol by hanging drop vapor diffusion, respectively, using a 1:1 protein:reservoir to crystallization buffer ratio at 20°C. Crystals were frozen in liquid nitrogen, and diffraction data were collected at 100 K and at the wavelength of 1.0 Å at Elettra Synchotron (Trieste, Italy). The diffraction data for HaNLP3 was processed to 2.0 Å resolution and to 1.95 Å resolution for NLP_Pya_^P41A, D44N, N48E^. Data were processed with XDS [16]. The crystal structures were solved using the symmetry of the space group P2_1_2_1_2_1_ for HaNLP3 and P4_1_2_1_2 for NLP_Pya_^P41A, D44N, N48E^ by molecular replacement using the NLP_Pya_ structure (PDB ID 3GNU) in PHASER [36] as a search model. Initial protein models were constructed with PHENIX Autobuild [37] and refined by iterative cycles of manual model building in Coot [38] and phenix.refine [17]. Structural data were visualized with Pymol. The coordinates have been deposited in the RCSB Protein Data Bank (HaNLP3: 6QBE, NLP_Pya_^P41A, D44N, N48E^: 6QBD).

### Molecular dynamics (MD) simulations

Starting points of our MD simulations were the crystal structures of NLP_Pya_ (PDB ID 3GNU) and MpNEP2 (PDB ID 3ST1) together with those of HaNLP3 (PDB ID 6QBE) and NLP_Pya_^P41A, D44N, N48E^ (PDB ID 6QBD) solved in this work. The HaNLP3 variants 1, 2 and 3 models were built by introducing the mutations into the crystal structure of HaNLP3 using the *t-leap* module of Ambertools 16 [39].

The protonation states of ionizable residues were assigned on the basis of the Propka program [40]. All proteins were considered in their ligand- and metal-free form. The heptapetide motif, which is involved in metal binding, is conserved in both NLP_Pya_ and HaNLP3 (as well as in all other simulated systems). It is likely that ion binding will similarly affect the dynamical properties of all investigated systems.

The topologies were built with Amber parm99-SB force field [41] using the Ambertools 16 module of AMBER program [39]. The system was solvated by adding a layer of 10 Å of TIP3P water molecules in each direction [42], leading to a total of 31639 atoms for NLP_Pya_, 33113 for HaNLP3, 33335 atoms of HaNLP3 variant 1, 26888 atoms for MpNEp2 and 31641 for NLP_Pya_^P41A, D44N, N48E^. Na^+^ ions were added to achieve charge neutrality using ion parameters [43].

The topologies were later converted in a GROMACS format using the software acpype. MD simulations were performed with GROMACS 5.0.4 [44]. An integration time step of 2 fs was used, and all covalent bonds, including hydrogen atoms, were constrained with the LINCS algorithm. The Particle Mesh Ewald algorithm [45] was used to account for electrostatic interactions. Simulations were performed in the isothermal-isobaric NPT ensemble at a temperature of 300 K under control of a velocity-rescaling thermostat [46] and Parrinello Rhaman barostat [47]. Preliminary energy minimizations were done with the steepest descent algorithm. The systems were then thermalized to the target temperature of 300 K during 5 ns of MD run. Finally, the NLP_Pya_, NLP_Pya_^P41A, D44N, N48E^, MpNEP2, HaNLP3 and its variants 1, 2, and 3 were equilibrated for 1 μs each, leading to a cumulative simulation time of 7 μs. The initial 200 ns of each simulation were not considered for the analysis.

Principal component analysis (PCA) was employed to extract the ‘essential movements’ of the proteins from the MD simulation trajectory. In this analysis, the mass-weighted covariance matrix of the C_α_ atoms of the protein was initially built from the atoms position vectors. A RMS-fit to the starting configuration of the MD simulation was performed to remove the rotational and translational motions. Once diagonalized, the covariance matrix leads to a set of orthogonal eigenvectors (principal components) and their corresponding eigenvalues. The eigenvectors with the largest eigenvalues correspond to the most relevant motions. Thus, the first principal component (PC1) is representative of the largest amplitude motion of the protein and this is usually referred to as “essential dynamics”. The Normal Mode Wizard plugin of the Visual Molecular Dynamics (VMD) molecular visualization program has been used to visualize the essential dynamics and to draw arrows highlighting the motion [48].

The correlation matrices, based on the Pearson’s correlation coefficients, were then employed to identify the coupling of the motions between each couple of residues along the MD trajectory. The values of the coefficients range from a value of -1, which indicates a totally anti-correlated motion, to a value of +1, which represents a linearly correlated lockstep motion [49, 50].

Root mean square fluctuations (RMSF), H-bond analysis, cross correlation matrices and PCA of the MD simulations trajectories were performed with the *cpptraj* module of Ambertools 16 [39].

## Supporting information

S1 Fig Stereo image of representative 2Fo-Fc (contoured at 1σ; gray mesh) and Fo-Fc (contoured at 3σ; red mesh, negative; green mesh, positive) electron densities of HaNLP3 polypeptide chain in the region between A107 and F114. The model of protein is shown in sticks.
(PDF)

S2 Fig Multiple sequence alignment of non-toxic HaNLP3 from *Hyaloperonospora arabidopsidis* and toxic oomycete type 1 NLPs from *Pythium aphanidermatum* (NLP_Pya_), *Moniliophthora perniciosa* (MpNEP1 and MpNEP2), *Phytophthora sojae* (PsojNIP), *Phythophthora infestans* (PiNPP1.1) and *Phythophthora parasitica* (PpNPP1). Pink, region between conserved disulfide bond type 1 NLPs; yellow, non-conserved disulfide bond in HaNLP3; cyan, L2 loop; green, NLP_Pya_ A127 and HaNLP3 W153 in Lc1 loop; red, L3 loop; blue, NLP_Pya_ D158, involved in sugar binding. Amino acids that were swapped between HaNLP3 and NLP_Pya_ are marked in bold.
(PDF)

S3 Fig Functional and biophysical characterization of NLP_Pya_ mutants. (A) Detection of necrosis-inducing ability of NLP_Pya_ mutants upon infiltration of proteins in tobacco leaf (2 dpi, 0.2 μM). (B) Circular dichroism of NLP_Pya_ mutants. (C) Representative melting curves of NLP_Pya_ mutants, measured in differential scanning fluorimetry assay. (D) Thermal stability of NLP_Pya_ mutants measured in thermofluor assays. Values are shown as means ± SD (n = 6).
(PDF)

S4 Fig Crystal structure of NLP_Pya_^P41A, D44N, N48E^ mutant. (A) Stereo image of representative 2Fo-Fc (contoured at 1σ; gray mesh) and Fo-Fc (contoured at 3σ; red mesh, negative; green mesh, positive) electron densities of NLP_Pya_^P41A, D44N, N48E^ polypeptide chain in the region between Ala69 and Gly76. (B) Overall crystal structure of NLP_Pya_^P41A, D44N, N48E^ (cyan, chain A; yellow, chain B) in comparison to apo-NLP_Pya_ (white). Mg^2+^ ions are displayed as purple spheres: (1), position of Mg^2+^ in apo-NLP_Pya_; (3), position of Mg^2+^ in NLP_Pya_^P41A, D44N, N48E^. Inset: Triple mutation site P41A, D44N and N48E. Amino acid residues are displayed as sticks. (**c**) Differences in positions of Mg^2+^ ion in apo-NLP_Pya_ (white), glucosamine-NLP_Pya_ (pink), polypeptide chain of NLP_Pya_ without bound hexose (light red) in the same asymmetric unit as glucosamine-NLP_Pya_ complex, and in chains A (cyan) and B (yellow) of NLP_Pya_^P41A, D44N, N48E^ crystal.
(PDF)

S5 Fig Dynamic properties of NLP_Pya_^P41A, D44N, N48E^. First column depicts graphical representation of root mean square fluctuation (RMSF) per residue projected on the most representative cluster of the protein (shown as tube) as extracted from the molecular dynamics (MD) trajectories. Colors from red to blue along with a decreasing diameter of the tube indicate a progressive RMSF decrease. In the second column we report the cross-correlation matrices. Correlation scores ranges from +1 (red) to -1 (blue). A positive sign of the map (blue color) indicates that residues move in a correlated manner (lockstep), otherwise, a negative value (red color) points to an anti-correlated motion between residues (opposite directions). White color shows that residue displacements are independent of each other. Third column shows essential dynamics of the protein. The latter is represented as cyan new cartoon and blue arrows indicate the direction and the amplitude of the motion.
(PDF)

S6 Fig Dynamic properties of MpNEP2. Left panel: a graphical representation of root mean square fluctuation (RMSF) per residue projected on the most representative cluster of the protein (shown as tube) as extracted from the MD trajectories. Colors from red to blue along with a decreasing diameter of the tube indicate a progressive RMSF decrease. Central panel: the cross-correlation matrix. Correlation scores range from +1 (red) to -1 (blue). A positive sign of the map (blue color) indicates that residues move in a correlated manner (lockstep), otherwise, a negative value (red color) points to an anti-correlated motion between residues (opposite directions). White color shows that residue displacements are independent of each other. Right panel: the essential dynamics of the protein. The latter is represented as a blue cartoon and blue arrows indicate the direction and the amplitude of the motion.
(PDF)

S7 Fig Dynamic properties of HaNLP3 (A) variant 1, (B) variant 2, and (C) variant 3. Left panel depicts a graphical representation of root mean square fluctuation (RMSF) per residue projected on the most representative cluster of the protein (shown as tube) as extracted from the molecular dynamics (MD) trajectories. Colors from red to blue along with a decreasing diameter of the tube indicate a progressive RMSF decrease. In the middle panel we report the cross-correlation matrix. Correlation scores ranges from +1 (red) to -1 (blue). A positive sign of the map (blue color) indicates that residues move in a correlated manner (lockstep), otherwise, a negative value (red color) points to an anti-correlated motion between residues (opposite directions). White color shows that residue displacements are independent of each other. On the right panel, the essential dynamics of the three proteins. These are represented as magenta new cartoons and blue arrows indicate the direction and the amplitude of the motion.
(PDF)

S8 Fig Leaf necrosis of NLP_Pya_ and HaNLP3 variant 3. (PDF)

S1 Movie Essential dynamics of NLP_Pya_. (AVI)

S2 Movie Essential dynamics of HaNLP3. (AVI)

S1 Table List of NLP_Pya_ mutants used in this study. (PDF)

S2 Table Thermal stability of NLP_Pya_ mutants. Values are shown as means ± SD (n = 6).
(PDF)

S3 Table Hydrogen bonds analysis of loops lining the GIPC head group binding cavity of HaNLP3 as extracted of MD simulations trajectory of HaNLP3. Residues from 114 to 128 belong to L2, from 177 to 187 belong to L3, from 151 to 157 to Lc1, from 54 to 62 to Lc3. Persistency (%) refers to the time the H-bond is established with respect to the length of the trajectory.
(PDF)

S4 Table Hydrogen bonds analysis of loops lining the GIPC head group binding cavity as extracted of MD simulations trajectory of NLP_Pya_. Residues from 90 to 104 belong to L2, from 150 to 162 belong to L3, from 126 to 131 to Lc1, from 31 to 39 to Lc3. Persistency (%) refers to the time the H-bond is established with respect to the length of the trajectory.
(PDF)

S5 Table Hydrogen bonds analysis of a wider region of loops Lc3 and L1 as extracted from the MD trajectories of (**a**) NLP_Pya_, (**b**) HaNLP3, (**c**) MpNEP2, (**d**) mutant NLP_Pya_^P41A, D44N, N48E^ and (**e**) mutant HaNLP3-variant 1. Persistency (%) refers to the time H-bond is established with respect to the length of the trajectory.
(PDF)

S6 Table List of mutations in chimeric HaNLP3 variants 1, 2 and 3. (PDF)
