# Supplementary material for: Molecular basis for functional diversity among microbial Nep1-like proteins
Source: PLoS Pathog. 2019 Sep 3;15(9):e1007951. doi: 10.1371/journal.ppat.1007951 (PMC6743777; doi:10.1371/journal.ppat.1007951)
Supplement: S1 Fig — The model of protein is shown in sticks. (PDF) [file ppat.1007951.s001.pdf]

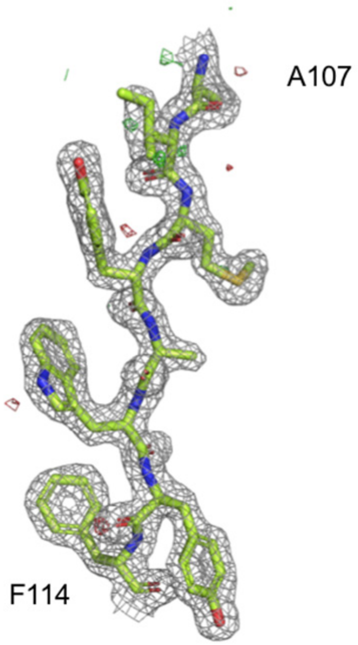

Supplementary Fig 1. Stereo image of representative 2Fo-Fc (contoured at 1σ; gray mesh) and Fo-Fc (contoured at 3σ; red mesh, negative; green mesh, positive) electron densities of HaNLP3 polypeptide chain in the region between A107 and F114. The model of protein is shown in sticks.
