# Supplementary material for: Molecular basis for functional diversity among microbial Nep1-like proteins
Source: PLoS Pathog. 2019 Sep 3;15(9):e1007951. doi: 10.1371/journal.ppat.1007951 (PMC6743777; doi:10.1371/journal.ppat.1007951)
Supplement: S3 Fig — (A) Detection of necrosis-inducing ability of NLPPya mutants upon infiltration of proteins in tobacco leaf (2 dpi, 0.2 μM). (B) Circular dichroism of NLPPya mutants. (C) Representative melting curves of NLPPya mutants, measured in differential scanning fluorimetry assay. (D) Thermal stability of NLPPya mutants measured in thermofluor assays. Values are shown as means ± SD (n = 6). (PDF) [file ppat.1007951.s003.pdf]

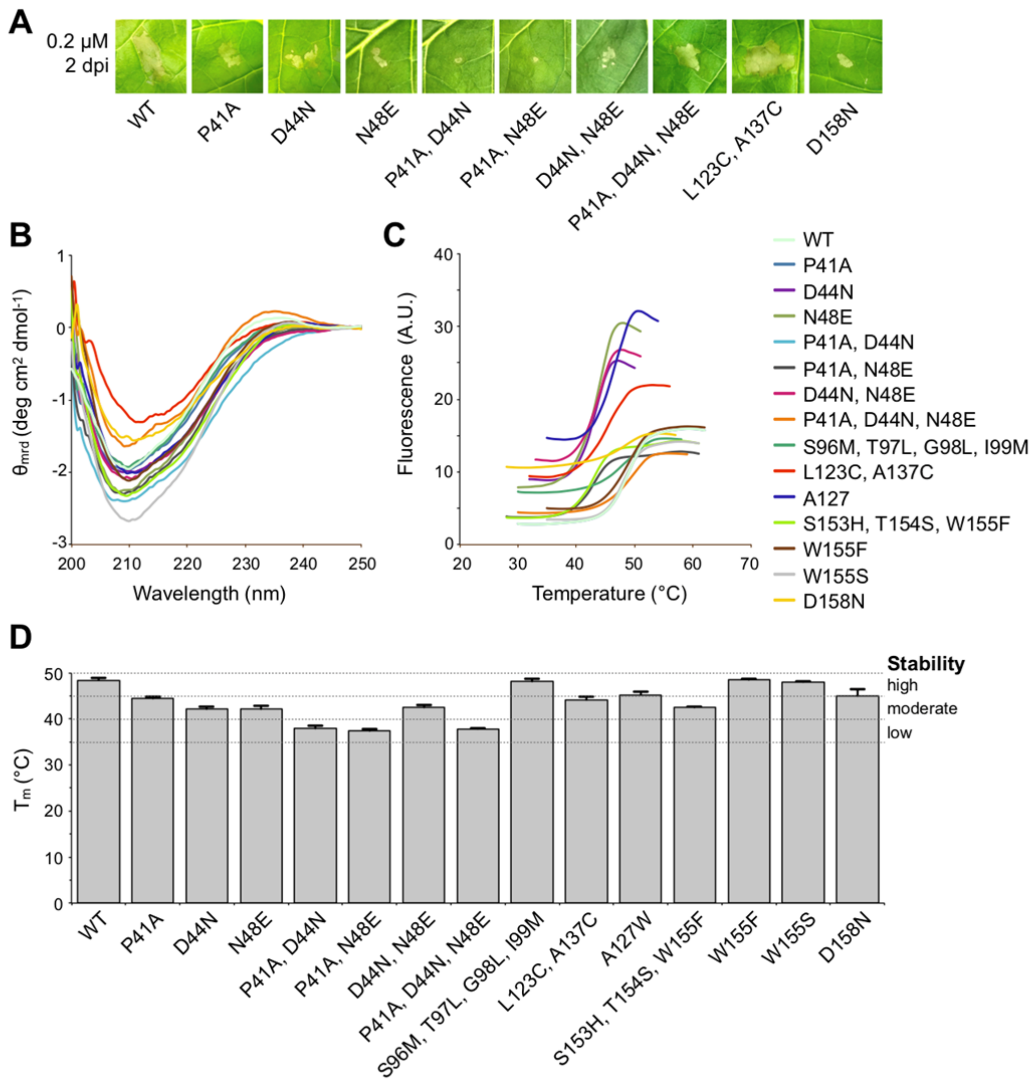

Supplementary Fig. 3. Functional and biophysical characterization of NLP_{Pya} mutants. (A) Detection of necrosis-inducing ability of NLP_{Pya} mutants upon infiltration of proteins in tobacco leaf (2 dpi, 0.2 μM). (B) Circular dichroism of NLP_{Pya} mutants. (C) Representative melting curves of NLP_{Pya} mutants, measured in differential scanning fluorimetry assay. (D) Thermal stability of NLP_{Pya} mutants measured in thermofluor assays. Values are shown as means ± SD (n=6).
