# Supplementary material for: Molecular basis for functional diversity among microbial Nep1-like proteins
Source: PLoS Pathog. 2019 Sep 3;15(9):e1007951. doi: 10.1371/journal.ppat.1007951 (PMC6743777; doi:10.1371/journal.ppat.1007951)
Supplement: S4 Fig — (A) Stereo image of representative 2Fo-Fc (contoured at 1σ; gray mesh) and Fo-Fc (contoured at 3σ; red mesh, negative; green mesh, positive) electron densities of NLPPyaP41A, D44N, N48E polypeptide chain in the region between Ala69 and Gly76. (B) Overall crystal structure of NLPPyaP41A, D44N, N48E (cyan, chain A; yellow, chain B) in comparison to apo-NLPPya (white). Mg2+ ions are displayed as purple spheres: (1), position of Mg2+ in apo-NLPPya; (3), position of Mg2+ in NLPPyaP41A, D44N, N48E. Inset: Triple mutation site P41A, D44N and N48E. Amino acid residues are displayed as sticks. (c) Differences in positions of Mg2+ ion in apo-NLPPya (white), glucosamine-NLPPya (pink), polypeptide chain of NLPPya without bound hexose (light red) in the same asymmetric unit as glucosamine-NLPPya complex, and in chains A (cyan) and B (yellow) of NLPPyaP41A, D44N, N48E crystal. (PDF) [file ppat.1007951.s004.pdf]

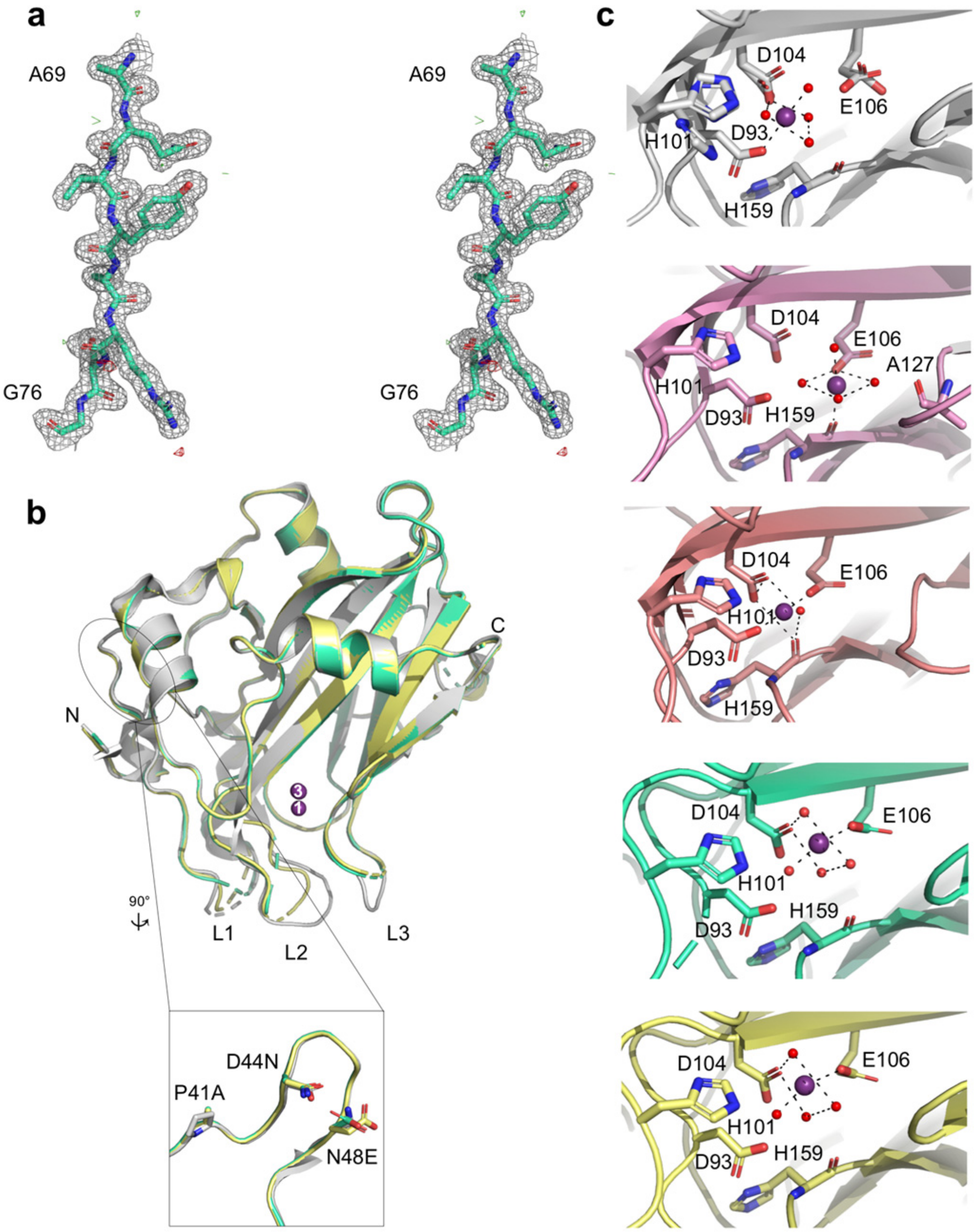

Supplementary Fig. 4. Crystal structure of $NLP_{Pya}^{P41A,\ D44N,\ N48E}$ mutant. (A) Stereo image of representative 2Fo-Fc (contoured at 1σ; gray mesh) and Fo-Fc (contoured at 3σ; red mesh, negative; green mesh, positive) electron densities of $NLP_{Pya}^{P41A,\ D44N,\ N48E}$ polypeptide chain in the region between Ala69 and Gly76. (B) Overall crystal structure of $NLP_{Pya}^{P41A,\ D44N,\ N48E}$ (cyan, chain A; yellow, chain B) in comparison to apo-NLP_{Pya} (white). Mg^{2+} ions are displayed as purple spheres: (1), position of Mg^{2+} in apo-NLP_{Pya}; (3), position of Mg^{2+} in $NLP_{Pya}^{P41A,\ D44N,\ N48E}$. Inset: Triple mutation site P41A, D44N and N48E. Amino acid residues are displayed as sticks. (**c**) Differences in positions of Mg^{2+} ion in apo-NLP_{Pya} (white), glucosamine-NLP_{Pya} (pink), polypeptide chain of NLP_{Pya} without bound hexose (light red) in the same asymmetric unit as glucosamine-NLP_{Pya} complex, and in chains A (cyan) and B (yellow) of $NLP_{Pya}^{P41A,\ D44N,\ N48E}$ crystal.
