# Supplementary material for: Molecular basis for functional diversity among microbial Nep1-like proteins
Source: PLoS Pathog. 2019 Sep 3;15(9):e1007951. doi: 10.1371/journal.ppat.1007951 (PMC6743777; doi:10.1371/journal.ppat.1007951)
Supplement: S5 Fig — First column depicts graphical representation of root mean square fluctuation (RMSF) per residue projected on the most representative cluster of the protein (shown as tube) as extracted from the molecular dynamics (MD) trajectories. Colors from red to blue along with a decreasing diameter of the tube indicate a progressive RMSF decrease. In the second column we report the cross-correlation matrices. Correlation scores ranges from +1 (red) to -1 (blue). A positive sign of the map (blue color) indicates that residues move in a correlated manner (lockstep), otherwise, a negative value (red color) points to an anti-correlated motion between residues (opposite directions). White color shows that residue displacements are independent of each other. Third column shows essential dynamics of the protein. The latter is represented as cyan new cartoon and blue arrows indicate the direction and the amplitude of the motion. (PDF) [file ppat.1007951.s005.pdf]

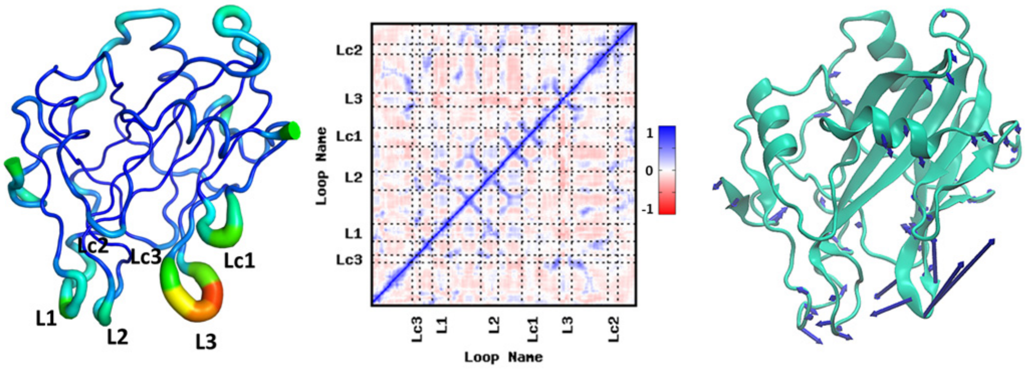

Supplementary Fig. 5. Dynamic properties of $NLP_{Pya}^{P41A,\ D44N,\ N48E}$. First column depicts graphical representation of root mean square fluctuation (RMSF) per residue projected on the most representative cluster of the protein (shown as tube) as extracted from the molecular dynamics (MD) trajectories. Colors from red to blue along with a decreasing diameter of the tube indicate a progressive RMSF decrease. In the second column we report the cross-correlation matrices. Correlation scores ranges from +1 (red) to -1 (blue). A positive sign of the map (blue color) indicates that residues move in a correlated manner (lockstep), otherwise, a negative value (red color) points to an anti-correlated motion between residues (opposite directions). White color shows that residue displacements are independent of each other. Third column shows essential dynamics of the protein. The latter is represented as cyan new cartoon and blue arrows indicate the direction and the amplitude of the motion.
