# Supplementary figures and images for: Molecular basis for functional diversity among microbial Nep1-like proteins
Source: PLoS Pathog. 2019 Sep 3;15(9):e1007951. doi: 10.1371/journal.ppat.1007951 (PMC6743777; doi:10.1371/journal.ppat.1007951)

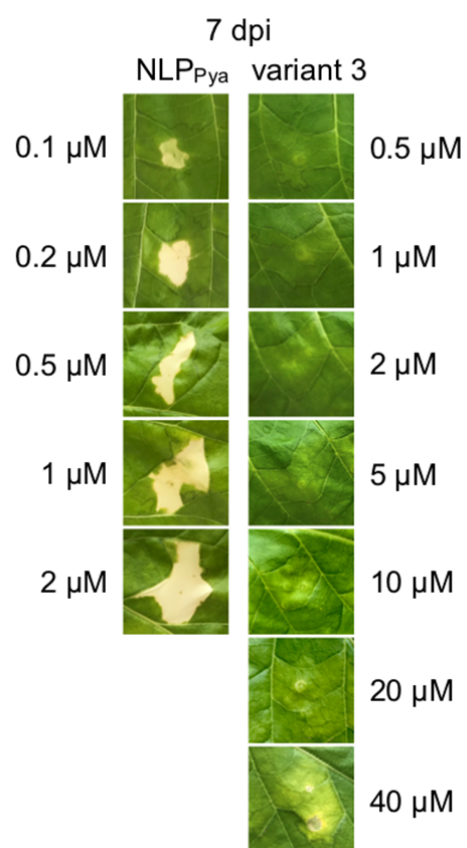

**Supplementary Fig. 8.** Leaf necrosis of NLP<sub>Pya</sub> and HaNLP3 variant 3.

Supplement: S8 Fig — (PDF) [file ppat.1007951.s008.pdf]
