# Supplementary material for: Molecular basis for functional diversity among microbial Nep1-like proteins
Source: PLoS Pathog. 2019 Sep 3;15(9):e1007951. doi: 10.1371/journal.ppat.1007951 (PMC6743777; doi:10.1371/journal.ppat.1007951)
Supplement: S1 Table — (PDF) [file ppat.1007951.s011.pdf]

| HaNLP3 sequence | Region | $NLP_{Pya}$ mutants |
|---|---|---|
| A64<br>N67<br>E71 | Between conserved disulfide bond | $NLP_{Pya}^{P41A}$<br>$NLP_{Pya}^{D44N}$<br>$NLP_{Pya}^{N48E}$<br>$NLP_{Pya}^{P41A,\ D44N}$<br>$NLP_{Pya}^{P41A,\ N48E}$<br>$NLP_{Pya}^{D44N,\ N48E}$<br>$NLP_{Pya}^{P41A,\ D44N,\ N48E}$ |
| M120<br>L121<br>L122<br>M123 | L2 loop | $NLP_{Pya}^{S96M,\ T97L,\ G98L,\ I99M}$ |
| C149<br>C163 | Non-conserved disulfide bond | $NLP_{Pya}^{L123C,\ A137C}$ |
| W153 | Lc1 loop | $NLP_{Pya}^{A127W}$ |
| H179<br>S180<br>F181 | L3 loop | $NLP_{Pya}^{W155F}$<br>$NLP_{Pya}^{W155S}$<br>$NLP_{Pya}^{S153H,\ T154S,\ W155F}$ |
| N184 | Phosphate binding | $NLP_{Pya}^{D158N}$ |

**Supplementary Table 1.** List of $NLP_{Pya}$ mutants used in this study.
