# Supplementary material for: Molecular basis for functional diversity among microbial Nep1-like proteins
Source: PLoS Pathog. 2019 Sep 3;15(9):e1007951. doi: 10.1371/journal.ppat.1007951 (PMC6743777; doi:10.1371/journal.ppat.1007951)
Supplement: S2 Table — Values are shown as means ± SD (n = 6). (PDF) [file ppat.1007951.s012.pdf]

NLP_{Pya} mutants	T_m (°C)
NLP_{Pya} wt	48.4 ± 0.4
NLP_{Pya}^{P41A}	44.4 ± 0.4
NLP_{Pya}^{D44N}	42.1 ± 0.6
NLP_{Pya}^{N48E}	42.2 ± 0.7
$NLP_{Pya}^{P41A, D44N}$	37.9 ± 0.6
$NLP_{Pya}^{P41A, N48E}$	37.3 ± 0.3
$NLP_{Pya}^{D44N, N48E}$	42.5 ± 0.6
$NLP_{Pya}^{P41A, D44N, N48E}$	37.8 ± 0.2
$NLP_{Pya}^{S96M, T97L, G98L, I99M}$	48.1 ± 0.6
$NLP_{Pya}^{L123C, A137C}$	44.1 ± 0.6
NLP_{Pya}^{A127W}	45.3 ± 0.6
NLP_{Pya}^{W155F}	48.5 ± 0.3
NLP_{Pya}^{W155S}	48.0 ± 0.2
$NLP_{Pya}^{S153H, T154S, W155F}$	42.5 ± 0.2
NLP_{Pya}^{D158N}	44.0 ± 1.4

Supplementary Table 2. Thermal stability of NLP_{Pya} mutants. Values are shown as means ± SD (n=6).
