# Supplementary material for: Molecular basis for functional diversity among microbial Nep1-like proteins
Source: PLoS Pathog. 2019 Sep 3;15(9):e1007951. doi: 10.1371/journal.ppat.1007951 (PMC6743777; doi:10.1371/journal.ppat.1007951)
Supplement: S3 Table — Residues from 114 to 128 belong to L2, from 177 to 187 belong to L3, from 151 to 157 to Lc1, from 54 to 62 to Lc3. Persistency (%) refers to the time the H-bond is established with respect to the length of the trajectory. (PDF) [file ppat.1007951.s013.pdf]

Acceptor	Donor	Persistency (%)
ASP_117@Od2	HIS_185@Hd1	67
ASP_55@O	TYR_177@H	66
HIS_185@Ne2	THR_58@Hg1	62
ASP_128@Od1	TYR_177@HH	57
GLU_178@O	ALA_186@H	53
TYR_177@O	ASN_57@H	51
ASN_184@O	SER_180@H	46
THR_58@Og1	HIS_179@H	45
ASP_117@Od2	HIS_185@H	34
SER_180@O	ASN_184@H	26
GLU_178@O	SER_180@Hg	24
ASP_117@Od1	HIS_185@H	23
LEU_187@O	THR_151@Hg1	20
ASN_184@O	SER_180@Hg	19
ALA_186@O	GLU178@H	16

Supplementary Table 3. Hydrogen bonds analysis of loops lining the GIPC head group binding cavity of HaNLP3 as extracted of MD simulations trajectory of HaNLP3. Residues from 114 to 128 belong to L2, from 177 to 187 belong to L3, from 151 to 157 to Lc1, from 54 to 62 to Lc3. Persistency (%) refers to the time the H-bond is established with respect to the length of the trajectory.
