# Supplementary material for: Molecular basis for functional diversity among microbial Nep1-like proteins
Source: PLoS Pathog. 2019 Sep 3;15(9):e1007951. doi: 10.1371/journal.ppat.1007951 (PMC6743777; doi:10.1371/journal.ppat.1007951)
Supplement: S4 Table — Residues from 90 to 104 belong to L2, from 150 to 162 belong to L3, from 126 to 131 to Lc1, from 31 to 39 to Lc3. Persistency (%) refers to the time the H-bond is established with respect to the length of the trajectory. (PDF) [file ppat.1007951.s014.pdf]

Acceptor	Donor	Persistency (%)
GLY_162@O	SER_150@H	79
ASP_158@O	THR_154@Hg1	73
ASP_158@O	THR_154@H	71
TYR_151@O	VAL_34@H	67
LYS_152@O	GLU_160@H	57
ASP_32@O	SER_150@Hg	53
SER_150@O	GLY_162@H	49
ASN_35@Od1	SER_153@H	43
ASP_32@O	TRY_151@H	33
GLU_160@Oe1	HIS_128@H	25
LEU_157@O	HIS_159@Hd1	25
GLU_160@O	LYS_152@H	22
HIS_159@O	HIS_128@He2	20
LEU_161@O	LYS_132@Hz1	18
GLU_160@Oe2	LYS_132@Hz2	17
GLU_160@Oe2	LYS_132@Hz3	15
LEU_161@O	LYS_132@Hz3	14

Supplementary Table 4. Hydrogen bonds analysis of loops lining the GIPC head group binding cavity as extracted of MD simulations trajectory of NLP_{Pya}. Residues from 90 to 104 belong to L2, from 150 to 162 belong to L3, from 126 to 131 to Lc1, from 31 to 39 to Lc3. Persistency (%) refers to the time the H-bond is established with respect to the length of the trajectory.
