# Supplementary material for: Molecular basis for functional diversity among microbial Nep1-like proteins
Source: PLoS Pathog. 2019 Sep 3;15(9):e1007951. doi: 10.1371/journal.ppat.1007951 (PMC6743777; doi:10.1371/journal.ppat.1007951)
Supplement: S5 Table — Hydrogen bonds analysis of a wider region of loops Lc3 and L1 as extracted from the MD trajectories of (a) NLPPya, (b) HaNLP3, (c) MpNEP2, (d) mutant NLPPyaP41A, D44N, N48E and (e) mutant HaNLP3-variant 1. Persistency (%) refers to the time H-bond is established with respect to the length of the trajectory. (PDF) [file ppat.1007951.s015.pdf]

a

Acceptor	Donor	Persistency (%)
ALA_42@O	SER_50@H	72
ASN_48@O	ASP_44@H	47
ASP_44@O	GLY_47@H	47
ASP_44@Od2	ASN_48@H	40
ASP_44@Od2	ASN_48@Hd22	36
ASP_44@Od1	ASN_48@Hd22	36
ASP_44@Od1	ASN_48@H	33
ASP_44@Od2	GLN_46@H	24
ASP_44@Od1	GLN_46@H	20
ASP_44@Od1	GLN_46@He22	5

b

Acceptor	Donor	Persistency (%)
ALA_65@O	SER_73@H	69
GLU_71@O	ASN_67@H	62
ASN_67@O	GLY_70@H	46
ASN_67@Od1	GLU_69@H	26
ASN_67@Od1	GLU_71@H	20
SER_73@Og	GLY_75@H	3
GLU_71@Oe2	THR_72@H	3
GLU_71@Oe1	THR_72@H	2
ASN_67@Od1	ALA_68@H	2
GLU_69@Oe1	ASN_67@Hd21	1

c

Acceptor	Donor	Persistency (%)
ALA_57@O	SER_65@H	83
ASP_59@O	GLY_62@H	42
ASP_59@Od2	ASN_63@Hd22	35
ASP_59@Od1	ASN_63@Hd22	31
ASN_63@O	ASP_59@H	26
ASP_59@Od1	ASN_63@H	19
ASP_59@Od2	ASN_63@H	17
ASP_59@Od2	GLN_61@H	11
ASP_59@Od1	GLN_61@H	11
ASP_59@Od2	GLN_61@He21	7

d

Acceptor	Donor	Persistency (%)
ALA_42@O	SER_50@H	78
ASN_44@Od1	GLU_48@H	60
GLU_48@O	ASN_44@H	48
ASN_44@O	GLY_47@H	45
ASN_44@Od1	GLN_46@H	19
SER_50@Og	GLY_52@H	6
GLU_48@Oe1	ASN_44@Hd21	6
GLN_46@Oe1	GLN_46@H	6
GLU_48@Oe2	ASN_44@Hd21	4
GLU_48@Oe1	GLN_46@He22	2

e

Acceptor	Donor	Persistency (%)
ALA_65@O	SER_73@H	68
GLU_71@O	ASN_67@H	61
ASN_67@O	GLY_70@H	46
ASN_67@Od1	GLU_69@H	26
ASN_67@Od1	GLU_71@H	19
GLU_71@Oe1	THR_72@H	4
SER_73@Og	GLY_75@H	4
GLU_71@Oe2	THR_72@H	4
GLU_71@Oe2	GLU_69@H	3

Supplementary Table 5. Hydrogen bonds analysis of a wider region of loops Lc3 and L1 as extracted from the MD trajectories of (**a**) NLP_{Pya}, (**b**) HaNLP3, (**c**) MpNEP2, (**d**) mutant $NLP_{Pya}^{P41A,\ D44N,\ N48E}$ and (**e**) mutant HaNLP3-variant 1. Persistency (%) refers to the time H-bond is established with respect to the length of the trajectory.
