# Supplementary material for: Molecular basis for functional diversity among microbial Nep1-like proteins
Source: PLoS Pathog. 2019 Sep 3;15(9):e1007951. doi: 10.1371/journal.ppat.1007951 (PMC6743777; doi:10.1371/journal.ppat.1007951)
Supplement: S6 Table — (PDF) [file ppat.1007951.s016.pdf]

Mutations	Region	Variant 1	Variant 2	Variant 3
M120S<br>L121T<br>L122G<br>M123I	L2 loop	No	Yes	Yes
H179S<br>S180T<br>F181W	L3 loop	Yes	No	Yes
W153A	Lc1 loop	Yes	Yes	Yes
N184D	sugar binding/<br>phosphate binding	Yes	Yes	Yes
N188D<br>N214D	Glycosylation	Yes	Yes	Yes

Supplementary Table 6. List of mutations in chimeric HaNLP3 variants 1, 2 and 3.
