# Supplementary material for: Molecular basis for functional diversity among microbial Nep1-like proteins
Source: PLoS Pathog. 2019 Sep 3;15(9):e1007951. doi: 10.1371/journal.ppat.1007951 (PMC6743777; doi:10.1371/journal.ppat.1007951)
Supplement: S2 Fig — Pink, region between conserved disulfide bond type 1 NLPs; yellow, non-conserved disulfide bond in HaNLP3; cyan, L2 loop; green, NLPPya A127 and HaNLP3 W153 in Lc1 loop; red, L3 loop; blue, NLPPya D158, involved in sugar binding. Amino acids that were swapped between HaNLP3 and NLPPya are marked in bold. (PDF) [file ppat.1007951.s002.pdf]

```
HaNLP3      RNDYVQEEKQQQLQEPLDGQWKPTTTGHDAIVPFSEPKPVTI-SEKAGVKFKPLLDVN   57
NLPPya      -----------------------AVINHDAVPVWPQPEPADA-TQALAVRFKPQLDVV   34
MpNEP1      ----------------------PHQLPHDQIAKFPKSG------GPLETQYQPALHIG   30
MpNEP2      ----------------------GTVMDHDKIAKLPASG------SPLETKFQPQLHIG   30
PsojNIP     -----------------------SVINHDQVVPFTQPTPTTA-LQQAAVKYKPQIHIS   34
PiNPP1.1    -----------------------DVISHDAVIPFAQPTTTTTTEQKAGIKFKPQIHIS   35
PpNPP1      -----------------------DVISHDAVVPFAQPTAT-TTEQKAGVKFKPQIHIS   34
                                       ** :                  :::* :.:

HaNLP3      TGCAPYAAVNAEGETSGGLQTSGDPESGCRGSKYGSQVYGRSTWYNDVWAIMYAWYFP   115
NLPPya      NGCQPYPAVDPQGNTSGGLKPSGSQAAACRDMS-KAQVYSRSGTYNGYYAIMYSWYMP   91
MpNEP1      NGCHSYPAVDVDGNWSGGLKPTGAPSAACKDTS-KAQTYVRSATFQGKTALVYAWYMP   87
MpNEP2      NGCHSYPAVDAQGNWSGGLKPTGAPSAACKDTS-KAQTYVRSATFQGKTALVYAWYMP   87
PsojNIP     NGCHPYPAVDNNGNTSGGLNPTGSESAGCKGSGYGTQIYGRAVKYQGVYAFMYSWYMP   92
PiNPP1.1    NGCHPYPAVDADGNTSGGLNPTGSSSAGCKGSGYGSQIYGRVATYNGVFAIMYSWYFP   93
PpNPP1      NGCHPYPAVDANGNTSGGLKPTGSSSAGCKGSGYGSQVYGRVATYNGVYAIMYSWYFP   92
            .**  * **: :*: ****: :*   :.*:.    :* * *   ::.  *::*:**:*

HaNLP3      KDSPMLLMGHRHDWENVVVFINDPDEVEPTILGCSTSWHSGYIKYAPCPTDSINGSSV   173
NLPPya      KDSPSTGIGHRHDWENVVVWLDNAA--SANIVALSASAHSGYKKSFPADKSYLDGITA   147
MpNEP1      KDEISTGIGHRHDWEGAVVFLNSDT---QQIDGVAASAHGKWRKYPNPGGANIDDTHV   142
MpNEP2      KDEISTGIGHRHDWEGAVVFLNSDT---QQIDGVAASAHGKWRKYPNPGGANIDDTHV   142
PsojNIP     KDETLTGLGHRHDWEACVVWVDDIAASSPKIVALSASAHSGYNKYYPPSSSYFSGNSA   150
PiNPP1.1    KDSPLTGLGHRHDWEHVVVWVDDIKLNSPSVIAVSPSAHSGYNIYHPPESNTIDGYST   151
PpNPP1      KDSPVTGLGHRHDWEHVVVWVDDIKLDSPSIIAVSPSAHSGYNIYYPPESNTIDGYSA   150
            **.    :*******  **:::.       : . : * *. :          :..  .

HaNLP3      MIKYEHSFP-LNHALNITKDAGAYQDLIMWHQMPDLARRALN-DTDFGKAITPMNDLN   229
NLPPya      KISYKSTWP-LDHELGFTTSAGKQQPLIQWEQMTQAARDALE-STDFGNANVPFKS-N   202
MpNEP1      KLQYSAEPVINSHALDLTDKGGDLPTLASWEGMGADARAAINERSHWGDANPPIADSL   200
MpNEP2      KLQYSAEPVINSHALDLTDKGGDLPTLASWEGMGADARAAINERSHWGDANPPIADSL   200
PsojNIP     KIDYSSSYVVINHALSATSTAGETQPLIMWDQLTDAARRALE-DTDFGDANVPFKDAN   207
PiNPP1.1    KVDYSSSWLVINHALDSTTDAGETQNLIMWDQLTDAARTALE-NTDFGDANVPMKDGN   208
PpNPP1      KVDYSSSWVVINHALDSTTDAGETQDLIMWDQLTDAARTALE-NTDFGDANVPMKDGN   207
             :.*.      .* *. *  .*    *  *. :   ** *::  :.:*.*  *: .

HaNLP3      FMEKIEAAWPFKTKKDGA   247
NLPPya      FQDKLVKAFFQ-------   213
MpNEP1      IDSSLSGAWMW-------   211
MpNEP2      IGSSLSGAWMW-------   211
PsojNIP     FQTKLGNAYYA-------   218
PiNPP1.1    FLTKVGNAYYA-------   219
PpNPP1      FLTKVGNAYYA-------   218
            :  .:  *:
```

Supplementary Fig. 2. Multiple sequence alignment of non-toxic HaNLP3 from *Hyaloperonospora arabidopsidis* and toxic oomycete type 1 NLPs from *Pythium aphanidermatum* (NLP_{Pya}), *Moniliophthora perniciosa* (MpNEP1 and MpNEP2), *Phytophthora sojae* (PsojNIP), *Phythophthora infestans* (PiNPP1.1) and *Phythophthora parasitica* (PpNPP1). Pink, region between conserved disulfide bond type 1 NLPs; yellow, non-conserved disulfide bond in HaNLP3; cyan, L2 loop; green, NLP_{Pya} A127 and HaNLP3 W153 in Lc1 loop; red, L3 loop; blue, NLP_{Pya} D158, involved in sugar binding. Amino acids that were swapped between HaNLP3 and NLP_{Pya} are marked in bold.
